# Electron diffraction characterization of nanocrystalline materials using a Rietveld-based approach. Part I. Methodology

**DOI:** 10.1107/S1600576722006367

**Published:** 2022-08-01

**Authors:** Ankur Sinha, Mauro Bortolotti, Gloria Ischia, Luca Lutterotti, Stefano Gialanella

**Affiliations:** a Department of Industrial Engineering, University of Trento, Via Sommarive 9, Trento, 38123, Italy; Ecole National Supérieure des Mines, Saint-Etienne, France

**Keywords:** transmission electron microscopy, instrumental broadening functions, Rietveld refinement

## Abstract

Transmission electron microscopy is a powerful experimental tool, very effective for the complete characterization of nanocrystalline materials by employing a combination of imaging, spectroscopy and diffraction techniques. Electron powder diffraction (EPD) pattern fingerprinting in association with chemical information from spectroscopy can be used to deduce the identity of the crystalline phases. Furthermore, EPD has similar potential to X-ray powder diffraction (XRPD) for extracting additional information regarding material specimens, such as microstructural features and defect structures. The aim of this paper is to extend a full-pattern fitting procedure, broadly used for analysing XRPD patterns, to EPD. The interest of this approach is twofold: in the first place, the relatively short times involved with data acquisition allow one to speed up the characterization procedures. This is a particularly interesting aspect in the case of metastable structures or kinetics studies. Moreover, the reduced sampling volumes involved with electron diffraction analyses can better reveal surface alteration layers in the analysed specimen which might be completely overlooked by conventional bulk techniques. The first step forward to have an effective application of the proposed methodology concerns establishing a reliable calibration protocol to take into correct account the instrumental effects and thus separate them from those determined by the structure, microstructure and texture of the analysed samples. In this paper, the methodology for determining the instrumental broadening of the diffraction lines is demonstrated through a full quantitative analysis based on the Rietveld refinement of the EPD. In this regard, a CeO_2_ nanopowder reference specimen has been used. The results provide indications also on the specific features that a good calibration standard should have.

## Abstract 2

Quantitative microstructural characterization of nanocrystalline materials based on Rietveld refinement of electron diffraction patterns has been used to explore sample characteristics. The electron microscope instrumental effects have been considered.

## 1. Introduction

The attractive properties of nanocrystalline materials have caused a significant growth in their production through bottom-up as well as top-down approaches (Iqbal *et al.*, 2012 ▸; Kumar *et al.*, 2018 ▸). Correspondingly, materials characterization protocols have been continuously updated in order to provide deeper insights into the physical and chemical properties of these materials. The key feature of this class of materials is their reduced grain size, with a relatively larger network of grain boundaries and relevant surface area (Gleiter, 1989 ▸). X-ray powder diffraction (XRPD) is an indispensable tool for the structure analysis of nanocrystalline materials. In the case of homogenous systems, a complete set of material features can be obtained from the XRPD patterns. One critical aspect might be the actual homogeneity of the specimens, as crucial local microstructural information could be missed. For this reason, electron diffraction (ED) in electron microscopes becomes a valuable option, although representativeness is still an issue (Weidenthaler, 2011 ▸).

Transmission electron microscopy (TEM)-based characterization is of particular interest when bulk quantities of the nanocrystalline materials are unavailable. The morphology and phase contrast of the particles from high magnification images, chemical information from energy-dispersive X-ray spectroscopy (EDXS) and electron energy loss spectroscopy, and phase identification and structure refinement based on selected area electron diffraction (SAED) patterns are very strong points in favour of TEM in the pursuit of a complete material characterization. Electrons have a much larger scattering cross section with matter than neutrons and X-rays, the typical wavelength of electrons is a hundred times lower, and, using suitable lens conditions, an electron beam can be focused to an extremely fine probe, even below 1 nm (Bendersky & Gayle, 2001 ▸). Moreover, in contrast to the X-ray radiation used for crystallography studies, which is sensitive only to electron density distribution, electrons are influenced by the electrostatic potential distribution in crystals (Zou, 2006 ▸). These features make ED suitable for probing extremely small volumes, with great sensitivity to changes in structure due to short-range ordering, lattice distortions, defects or the presence of secondary phases. Additionally, data acquisition for a wide interplanar spacing range in the case of TEM takes a few seconds. Contrarily, acquiring the XRD data typically takes from several minutes up to hours. In the case of a large number of randomly oriented crystallites, SAED patterns have the appearance of nested rings. This feature is analogous to Debye–Scherrer patterns as obtained from X-ray diffraction experiments.

However, a characteristic feature of electron diffraction is a strong coulombic interaction between the high-energy (usually 80–300 keV) electron beam of small coherence length and the sample, leading to dynamic scattering (Midgley & Eggeman, 2015 ▸; Cowley, 1995 ▸). For countering the constraints of the kinematical theory, which remains valid for smaller crystallite sizes only and high electron energy (Weirich *et al.*, 2000 ▸; Zuo *et al.*, 2019 ▸), in two-beam dynamical theory an approximation concerning the diffracted intensity is considered (Blackman & Thomson, 1939 ▸). In this context, structure analysis using a Rietveld refinement (Rietveld, 1967 ▸) has been conducted on SAED patterns of nanocrystalline materials: TiO_2_ (Weirich *et al.*, 2000 ▸, 2002 ▸; Boullay *et al.*, 2014 ▸), Mn_3_O_4_ (Boullay *et al.*, 2014 ▸), MnFe_2_O_4_ (Kim *et al.*, 2009 ▸), Al and α-MnS (Gemmi *et al.*, 2011 ▸), hy­droxy­apatite (Song *et al.*, 2012 ▸; Schlieper *et al.*, 2010 ▸), and ZnS–ZnO (Serafini *et al.*, 2017 ▸). In a sample containing more than one phase, diffraction peak overlap is a rather common issue, often associated with a significant broadening of the diffraction lines. In this case, Rietveld refinement for a complete microstructural characterization is especially suited, since the diffraction contributions of each phase can be effectively separated. This approach may in principle support a conventional TEM analysis. Depending on the diffraction contrast, the dark-field mode may fail to obtain high-resolution images of individual crystallites of any specific phase, if the relevant diffraction signal interferes with those of other phases, not to mention the unavoidable astigmatism limitations (Serafini *et al.*, 2017 ▸). Such problems pertaining to the microstructural characterization are amplified when aggregates of nanograins are present, thus worsening further interference effects (Dieckmann *et al.*, 2009 ▸).

It is desirable that the physical broadening, which contains microstructural information pertaining to the sample, has a greater impact on the observed patterns than the instrumental broadening (Balzar *et al.*, 2004 ▸; He *et al.*, 2018 ▸). This aspect has already been investigated extensively with reference to X-ray diffraction (XRD) (Scardi *et al.*, 1994 ▸; Ida & Toraya, 2002 ▸; Chateigner, 2013 ▸). If the role of the instrument in the broadening of the reflections is not taken into account, this may cause erroneous results in the evaluation of the sample characteristics. For instance, a 25% error in the measured crystallite size of a nanosized Cr_2_O_3_ sample was noted (Weidenthaler, 2011 ▸). Although electron-diffraction-based structural analysis has gained prominence over the years (Weirich *et al.*, 2002 ▸; Kim *et al.*, 2009 ▸), literature concerning standard procedures to calibrate a transmission electron microscope for its instrumental broadening contribution to ED data is relatively scarce (Boullay *et al.*, 2014 ▸). In this paper, a complete quantitative characterization based on the Rietveld refinement of EPD patterns of a nanocrystalline standard sample using *MAUD* (*Material Analysis Using Diffraction*; Lutterotti, 2010 ▸; Lutterotti *et al.*, 1997 ▸) was performed, which incorporates the dynamical effects for small crystals. We have demonstrated how the TEM instrumental broadening function can be measured following a sequence of steps in correlation with XRD. To make the approach robust and all inclusive, multiple diffraction patterns were collected under different TEM operating conditions: varying the camera length, selected area (SA) aperture and the second condenser lens (C2) current intensity. A comparative study of the diffraction patterns from these different operating conditions has been conducted to explore important aspects of electron diffraction from nanocrystalline materials.

## 2. Experimental

EPD data were collected on a STEM-EDXS Talos F200S (Thermofisher Scientific) instrument operated at 200 keV. The diffraction patterns were collected with different SA apertures: 800, 200 and 40 µm diameter. To prevent damage to the CCD detector, the transmitted beam was intercepted with a beam stopper.

A CeO_2_ (Alfa Aesar, 99.5% minimum rare earth oxide, 15–30 nm) nano-powder was used as a standard reference sample. This sample has been selected since CeO_2_ (cubic; space group 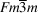 ) has relatively simple crystal structures and well defined chemical and physical properties. It features a narrow crystallite size distribution resulting in a diffraction line broadening that is independent of the crystallographic direction (isotropic sample). A small amount of the CeO_2_ nanopowder was dispersed in ethanol using ultrasonic mixing for 10 min. Thereafter, a few drops of the suspension were deposited onto a carbon-coated Cu (300 mesh) TEM grid.

X-ray powder diffraction data were collected on the same powder in reflection geometry using an Italstructures IPD3000 diffractometer; the instrument was equipped with a Cu-anode X-ray source coupled to a multilayer monochromator on the incident beam. Patterns were acquired through a 4096-channel curved position sensitive detector (Inel CPS120) covering 120° 2θ angular range (approximate channel resolution 0.03°) with an acquisition time of 30 min.

## 3. Methodology

### 3.1. Combined XRPD and EPD analyses

The observed diffraction profile (*h*) of a polycrystalline material is broadened owing to sample (*f*) as well as instrumental (*g*) effects. The combination of the two is represented by the convolution product (Scardi & Leoni, 2006 ▸): *h* = *f* ⊗ *g* + *b*. The term *b* is added to model the background. *g* is the convolution of the emission profile of the radiation and all instrumental aberrations, whether physical or geometric (Soleimanian & Aghdaee, 2008 ▸). An ideal standard material to be used for determining the instrumental peak shape function should not contribute to the line broadening substantially. Thus, the entire broadening of the diffraction pattern can be attributed to the instrument. Usually, a reference standard sample that is suitable for the XRD instrument is not appropriate for TEM, since larger crystallite sizes cause discontinuous and grainy electron diffraction ring patterns. TEM samples should also exhibit a thickness that is small enough to guarantee electron transparency, so that a part of the incoming beam is transmitted and forms the relevant diffraction pattern, instead of being fully absorbed or backscattered.

For X-ray powder diffraction, a reference sample with large isotropic crystallites and negligible microstrain is preferred, since in this way the instrumental contribution to the scattered intensity can be better estimated. In this regard, Y_2_O_3_ powder from Sigma Aldrich (99.99% trace metals basis) was adopted as a calibration standard after calcination at 1573 K for 24 h to remove any possible lattice strain. The peak broadening of the collected pattern was refined by setting to zero the size/strain broadening contribution of the sample and refining the instrumental function for the XRD instrument, *g*_XR_(*x*), in the form of the Caglioti function. Subsequently, the same instrument was used for a complete microstructural characterization, *f*_XR_(*x*), of the TEM standard sample as follows (Boullay *et al.*, 2014 ▸):

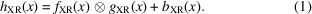

The complete output from the XRD data refinement *f*_XR_(*x*) was taken as the input for EPD analysis, ultimately leading to the determination of the TEM instrumental broadening function parameters, *g*_TEM_(*x*):

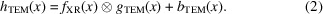

During this latter analysis, the *f*_XR_(*x*) parameters were kept constant and not refined.

### 3.2. EPD: data collection and analysis approach in *MAUD*

**Figure 1:**
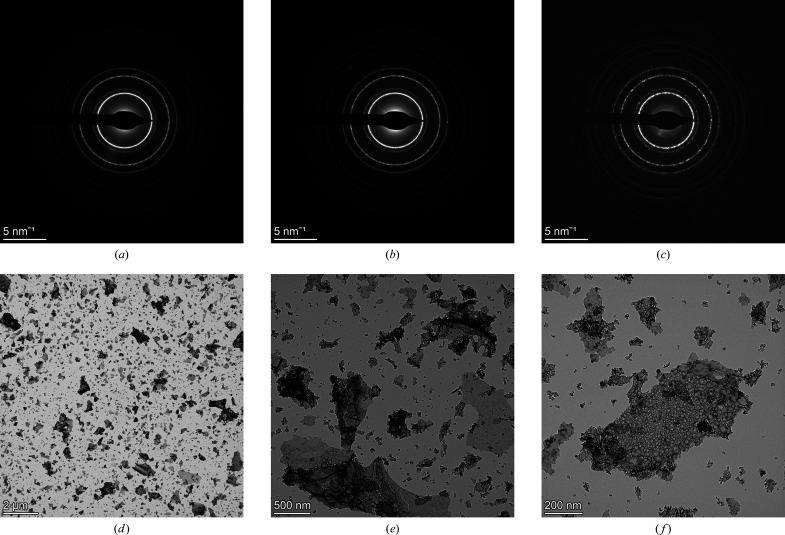
Diffraction patterns of the CeO_2_ standard sample, corresponding to camera length 844 mm acquired using (*a*) 800 µm SA aperture, (*b*) 200 µm SA aperture and (*c*) 40 µm SA aperture from the field of view in (*d*), (*e*) and (*f*), respectively.

We used three different SA apertures: 800, 200 and 40 µm. Corresponding to each of these apertures, we collected diffraction patterns at four different camera lengths: 1360, 1080, 844 and 658 mm. Thus, in total 12 diffraction patterns were analysed. For one particular camera length of 844 mm, Figs. 1 ▸(*a*)–1 ▸(*c*) show the diffraction patterns corresponding to the largest to the smallest apertures, respectively, from the fields of view in Figs. 1(*d*)–(*f*). There is a marked difference in the appearance of the patterns. In the 800 µm aperture diffractogram a negligible number of individual, brighter spots along the otherwise homogeneous diffraction rings are visible [Fig. 1 ▸(*a*)], whereas relatively more of these isolated spots are present in the pattern acquired with the 40 µm aperture [Fig. 1 ▸(*c*)]. This is an expected effect of the different statistics that different selected areas would induce.

**Figure 2:**
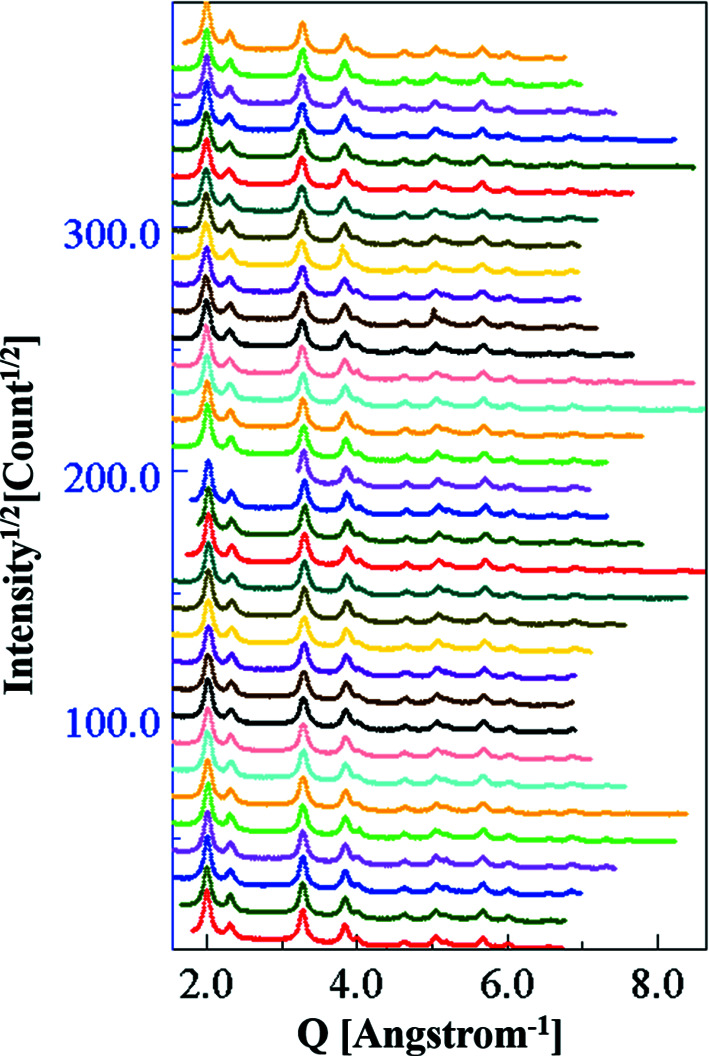
Diffractograms obtained from the azimuthal segmentation (10° sectors) of the SAED pattern. These were then integrated to obtain the 1D ED pattern. The data refer to the SA aperture 200 µm–CL 1080 mm combination.

Each SAED pattern was first imported into *MAUD* using the *ImageJ* (https://imagej.nih.gov/ij/) plug-in and the rings were segmented into 10° wide sectors. Fig. 2 ▸ shows the complete series of diffractograms generated through the azimuthal segmentation of the rings. Thirty-four diffractograms were obtained, after excluding the zero-signal region corresponding to the beam stopper. The multiple diffractograms resulting from this process were used as data files. These were integrated to form the final 1D plot of the ED data, which served as the starting reference set for the Rietveld refinement. Using this process, we could convert the 2D image to a 1D pattern with a low textural signature and also preserve the positions of the coordinates of the points originally present in the image (Ischia *et al.*, 2005 ▸; Lutterotti *et al.*, 2014 ▸). For the complete procedure, the reader is referred to the supplementary file that contains all the necessary steps.

It is necessary to have a good fit for the background, which is composed of the following main terms: inelastic scattering, incoherent multiple scattering and scattering from the amorphous supporting film, if any (Williams & Carter, 2009 ▸; Andrews *et al.*, 1967 ▸). For the present case, the background was fitted using a polynomial function of the fourth degree. The transmitted beam causes a sharp increase in the background intensity at low angles. Thus, an additional Gaussian peak was included for a better fit.

**Figure 3:**
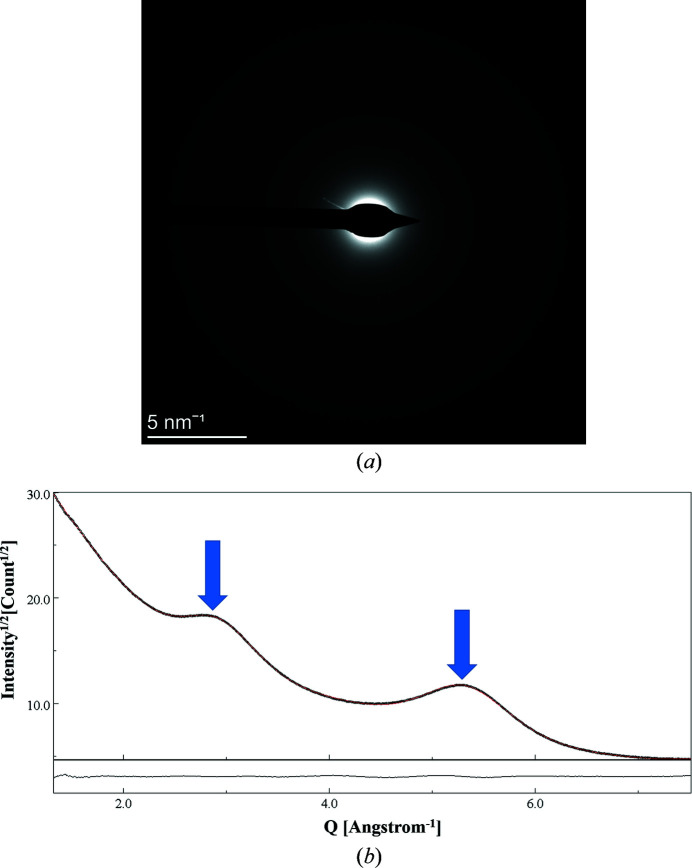
(*a*) Diffraction pattern from the naked Cu grid with the supporting C film. (*b*) Intensity profile from the C-supporting film with halos marked by two arrows. The data refer to the SA aperture 200 µm–CL 1080 mm combination. *Y* axis: sqrt(intensity); *X* axis: *Q* (Å)^−1^.

The Cu grid used had a supporting amorphous C film. Thus, the EPD patterns had also a diffused contribution from the scattering intensity coming from the C film (Kim *et al.*, 2009 ▸). This amorphous diffraction intensity profile, with two halos, is shown in Fig. 3 ▸. The intensity of these halos causes a mismatch between the experimental and the calculated profiles, which could lead to incorrect determination of the microstructural parameters. To account for these differences, the profiles of the amorphous diffraction patterns obtained from the naked Cu grid were also fitted separately. The intensities of the halos, along with their shape configurations, were added manually during the refinement of the CeO_2_ patterns.

The best solution is achieved by minimizing the weighted sum of the quadratic scattering values, *S_y_*, given by the equation

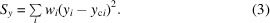

Here *y_i_* is the experimental and *y*_c*i*_ is the calculated intensity at the *i*th step; *w_i_* = 1/*y_i_* is the relevant weight. In *MAUD*-based analyses, the Rietveld refinement of the EPD pattern is performed using the electron atomic scattering factors from Peng *et al.* (1996 ▸).

A pseudo-Voigt function was used to model the Bragg peaks, since this function provides the closest match between the observed and the calculated data (Weirich *et al.*, 2002 ▸; Li, 2010 ▸). A pseudo-Voigt function is a linear combination of Lorentzian and Gaussian functions and is capable of describing a continuous variation in the line profile between the two component functions, to estimate better both crystallite size and strain line-broadening contributions (de Keijser *et al.*, 1983 ▸).

**Figure 4:**
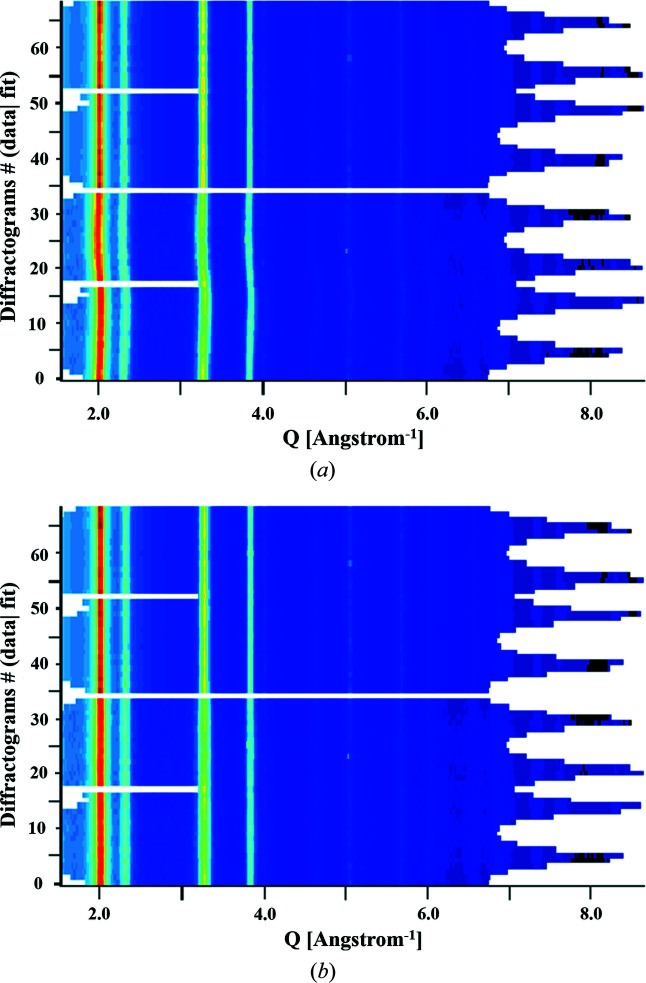
Two-dimensional multiplot of the calculated (upper part) and experimental (bottom part) profiles are displayed. In (*a*) we see an offset between the data and the fit, as well as ‘waviness’ in the experimental profile, which is corrected in (*b*) by accounting for both centring and elliptical errors. (*b*) has been plotted just after implementing the corrections and these refer to the SA aperture 200 µm–CL 1080 mm combination.

The starting refinement cycles were employed for adjusting the background using a fourth-degree polynomial and for refining the scaling factors. Subsequently, errors in the centring of the original 2D diffraction image and those caused by detector tilting, *i.e.* the detector not being perpendicular to the electron beam, were refined. This step leads to the correct transformation of the image coordinates, *i.e.* experimental points, into *Q* values (Lutterotti *et al.*, 2014 ▸). This has been demonstrated in Fig. 4 ▸: the aforementioned errors are visible in Fig. 4 ▸(*a*) but upon including the corrections are reduced in Fig. 4 ▸(*b*).

TEM manufacturers provide camera lengths (CLs) for different magnification steps. However, these values might be in error by up to 10% (Williams & Carter, 2009 ▸; McCaffrey & Baribeau, 1995 ▸). With the incorrect camera length, there would obviously be large variations in the diffracted peak positions of the calculated and experimental profiles. The variation in the camera length is mainly due to the following factors: the hysteresis present in the electromagnetic lenses, the position of the specimen within the objective lens, the electron beam convergence based on the condenser lens settings, and the focus conditions (Zuo *et al.*, 2019 ▸). For the determination of the ‘correct’ camera length, a possible method might be to calibrate multiple diffraction patterns obtained using different selected area apertures, for a specific camera length. If the variation observed for the different SA apertures is within specified limits, we can assume that the correct value of CL has been approached.

At the time of acquisition of the diffraction pattern, the standard sample must be at the eucentric height of the stage, the objective lens should be set to its standard focus and a representative region of the sample should be selected avoiding any possible contribution from the sustaining TEM grid, if present. The C2 current intensity variation can cause a change in the measured value of the camera length. However, in our experience, if the sample is placed at the eucentric height, the astigmatism of the condenser and objective lenses have been compensated, the image is focused with the help of fast Fourier transform, and diffraction astigmatism, if present during the acquisition of the diffraction pattern, has also been corrected, then we do not need to change the C2 current intensity for collecting diffraction patterns at different camera lengths for a particular setting. Any further adjustment, if required, upon changing the camera length is accomplished by just using the diffraction focus.

Thereafter, using the equation *Rd* = λ*L* (where *R* is the diffraction ring radius, *d* the crystal lattice spacing of the standard material, λ the wavelength of the electron at the operating accelerating voltage and *L* the corrected camera length), the corrected camera length can be determined.

**Figure 5:**
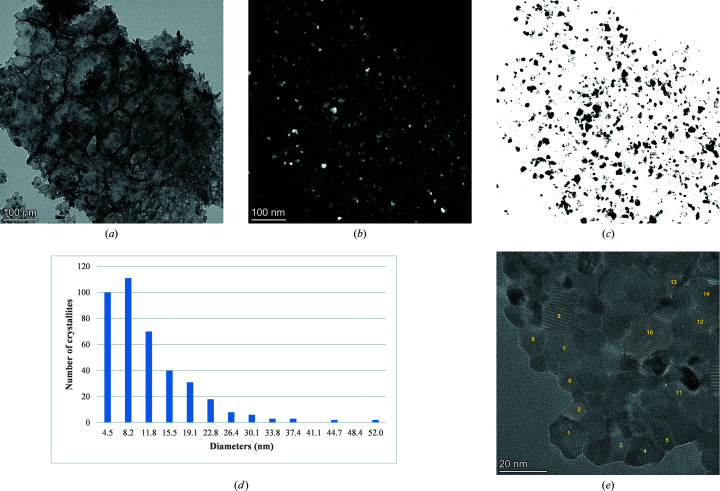
(*a*) BF micrograph of a CeO_2_ nanoparticle aggregate. (*b*) Corresponding DF micrograph. (*c*) Threshold of the DF micrograph shown in (*b*). (*d*) Histogram of the size distribution of crystallites, used to determine an average size of 135 Å. (*e*) High-magnification image with 14 marked crystallite domains that were clearly visible. These were used to estimate the average crystallite size.

To select the model better suited to describing the line broadening due to microstructural features, we analysed the XRD data using the simple ‘Delft’ model as well as ‘Popa LB’ (de Keijser *et al.*, 1982 ▸). The Delft model provided an average size of the crystallite of 124.9 (4) Å, which was closer to the average value of 128 Å obtained from the TEM BF image shown in Fig. 5(*e*) (see Section 4). The Popa LB model yielded an average crystallite size of 98 Å, thus showing a larger variance. Consequently, the Delft model was selected for all successive stages of the refinement.

Le Bail fitting is a faster and more efficient approach based on the extraction of the integrated intensities (with possible overlaps) of individual reflections from the diffraction data. This method does not necessarily need a structural model but rather constrains the angular positions of the reflections on the basis of space groups and initial unit-cell parameters (Le Bail *et al.*, 1988 ▸; Le Bail, 1989 ▸). The reflection intensities are initially set to arbitrary values and are evolved iteratively through the least-square procedure included in the Rietveld decomposition formula (Peterson, 2005 ▸). The Le Bail method provides an indication of the best profile fit that can be obtained. Also, the *R*_wp_ value in the Rietveld refinement, where structural aspects are taken into consideration, should approach the *R*_wp_ value from the Le Bail method (McCusker *et al.*, 1999 ▸), which was used for measuring *g*_TEM_(*x*) too.

### 3.3. Two-step calibration

As a next step, we wanted to validate the consistency of the proposed method. In this phase of the analysis, we fixed the parameters of the instrumental broadening function as obtained previously and determined the sample structural and microstructural characteristics *f*_TEM_(*x*), lattice parameter, crystallite size(s) and microstrain(s) using

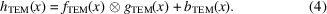

This step provided the opportunity to compare the sample characteristics obtained from XRPD, *f*_XR_(*x*), with those obtained from EPD, *f*_TEM_(*x*). In this ‘two-step calibration’ stage, we analysed the EPD with three different modes: the Le Bail method, the kinematical approximation and the Blackman two-wave dynamic correction (Blackman & Thomson, 1939 ▸).

The interactions between the electrons and atoms may result in multiple scattering. The issue with kinematical scattering is strong extinction because of which the integrated intensity is reduced, thus rendering the kinematical approximation suitable for very small particles and thin films only (Chen & Zuo, 2007 ▸; Cockayne, 2007 ▸). Horstmann & Meyer (1962 ▸) estimated that the dynamical scattering component is below 10% for ED patterns of polycrystalline Al crystals, smaller than 9 nm for electron-beam energies in the range 20–50 keV. Weirich *et al.* (2002 ▸) observed weak dynamical effects for 120 keV energy-filtered EPD collected on texture-free nanocrystalline anatase of average crystallite size 7 nm. Kim *et al.* (2009 ▸) noted that, for a 120 kV electron beam, the ratio of the kinematical to dynamical contributions towards the structure factor was approximately 1:1.5 for polycrystalline MnFe_2_O_4_ with 11 nm average crystal size. Luo *et al.* (2011 ▸) concluded that a small correction for the dynamical scattering (less than 3%) could improve the long-range order parameter in an Au_3_Fe_1−*x*_ alloy. Thus, it is favourable to analyse the EPD patterns by considering the Blackman two-beam correction, which provides the intensities of the reflections as a function of crystal thickness and electron wavelength. In *MAUD*, it is possible to include the full Blackman formulation with the usual approximations in the refinement (Boullay *et al.*, 2014 ▸). The dynamic intensity, *I*_d_, can be expressed in terms of the structure factor *F*_*hkl*_ as (Zuo *et al.*, 2019 ▸; Blackman & Thomson, 1939 ▸)

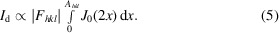

Here, *A*_*hkl*_ ≃ γλ|*F_hkl_* |*t*/*V*_c_. As fully discussed by Zuo *et al.* (2019 ▸), in these expressions, *t* is the thickness of the crystallites along the beam direction, *V*_c_ is the cell volume, λ is the electron wavelength, γ is the relativistic constant for the electron and *J*_0_(2*x*) is the zero-order Bessel function. If the value of the Bessel function is equal to 1, which is true when *A*_*hkl*_ has a small value, the dynamical intensity approaches the kinematical limit (Spence & Zuo, 1992 ▸). From this, the following expression for the dynamical intensity is derived:

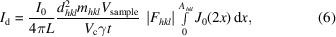

where *m*_*hkl*_ is the multiplicity of the reflection, *V*_sample_ is the sample volume, *L* is the camera length and *d*_*hkl*_ is the interplanar spacing.

However, for a large value of *A*_*hkl*_, the integral over the Bessel function approaches the value of 1/2. In this scenario, the diffracted intensity is found to be proportional to the structure factor amplitude, and not to its square, as predicted by the kinematical theory. For the kinematical theory, the following expression applies (Vainshtein, 1964 ▸):

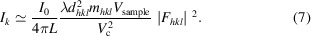

On these bases, the two-beam correction has been implemented in *MAUD* and other programs, like *PCED 2.0* (Li, 2010 ▸).

To explore the size and shape variations of the crystallites depending on the (*hkl*) planes, the Popa model (Popa, 1998 ▸) was considered. The latter gave a direct indication of the anisotropy present in the analysed sample. According to the Popa model for the anisotropic shape of a crystallite, the average radius 〈*R*_*hkl*_〉 for the Laue class *m*3*m* can be expressed as

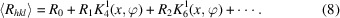

In the above convergent harmonic series with *R*_*i*_ coefficients, the first coefficient *R*_0_ refers to the average radius of the isotropic crystallite; *x* = cosϕ, where ϕ is the polar angle and φ is the azimuthal angle in an orthogonal coordinate system. The equation remains valid for any shape and size distribution of the crystallites and has been incorporated into *MAUD*. Thus, the (*hkl*) dependence of the diffraction line broadening can be determined. Since in this paper we have analysed CeO_2_, which has a cubic crystal structure, we can use the term ‘[*hkl*] direction’ with reference to the dimension of a crystallite perpendicular to the (*hkl*) crystallographic plane.

## 4. Results and discussion

### 4.1. Camera length calibration

The diffraction patterns collected with the standard CeO_2_ sample revealed a uniform and continuous distribution of the diffracted intensity along the rings (see Fig. 1 ▸). This signifies the absence of any preferred orientation in the sample, as confirmed through the Rietveld refinement. Indeed, a good fit was obtained without including any texture contribution available in the software package. This aspect can be taken as a further proof of the adequate quality of the selected CeO_2_ powder sample. For a comparative estimation of the average crystallite size, we considered a dark-field (DF) image of a particle cluster. Figs. 5 ▸(*a*) and 5 ▸(*b*) show the BF and DF images of a crystallite cluster, along with the *ImageJ*-analysed DF image [Fig. 5 ▸(*c*)] and the histogram [Fig. 5 ▸(*d*)] of the size distribution of the crystallites, with an average value of 135 Å. Fig. 5 ▸(*e*) is a high-resolution image of the CeO_2_ specimen, with selected numbered crystallites. These crystallites have an average size of 128 Å, as estimated using the *ImageJ* software.

**Figure 6:**
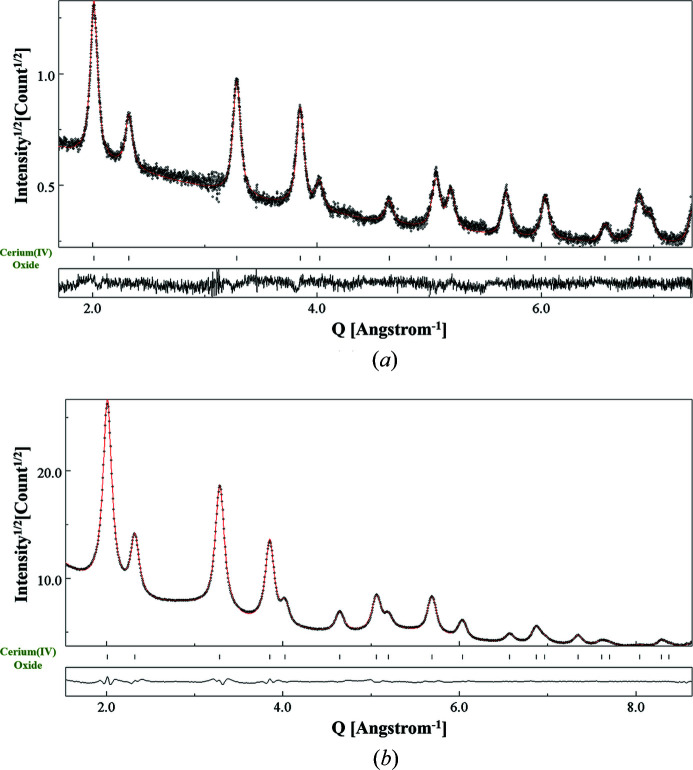
Results of the combined analysis of CeO_2_ nanopowders for (*a*) XRPD patterns considered to extract *f*_XR_(*x*), and (*b*) EPD fitted with input from (*a*) using a pattern-matching mode (Le Bail). (*b*) refers to the SA aperture 200 µm–CL 1080 mm combination. Dots: experimental intensity profile; red line: calculated profile. *Y* axis: sqrt(intensity); *X* axis: *Q* (Å)^−1^.

With reference to the XRPD and EPD profile fitting patterns shown in Fig. 6 ▸, the relevant calculated *Y*_calc_ (black dots) and experimental *Y*_exp_ (red dots) intensities along with the residual curve have been plotted. The refined XRD data are shown in Fig. 6 ▸(*a*). An average crystallite size of 124.9 (4) Å was obtained, including also the shape anisotropy. The reliability factor along with the lattice constant is given in Table 1 ▸. Fig. 6 ▸(*b*) corresponds to the profile fitting of the EPD data collected using 200 µm SA aperture and 1080 mm CL. In all tables and physical parameters reported in the paper, we adopted the standard criterion (for Rietveld refinement) to provide the decimal digits up to the value of the estimated standard deviation, as given by the algorithm.

**Figure 7:**
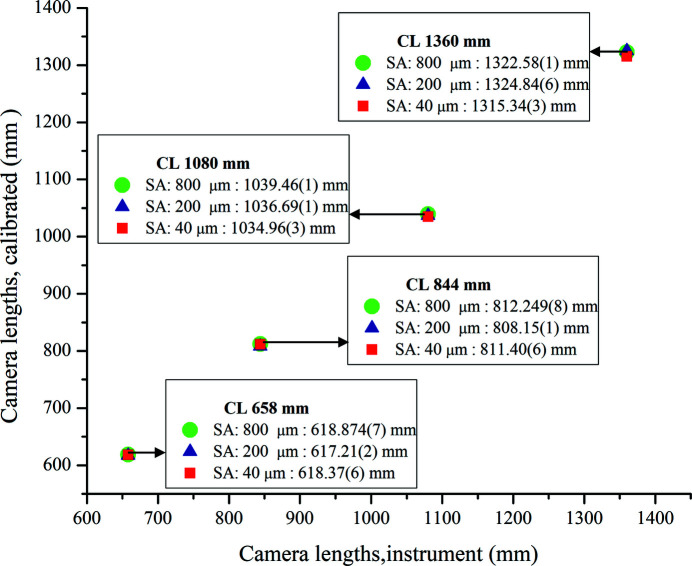
Variation from the instrumental nominal values amongst calibrated camera length values.

**Figure 8:**
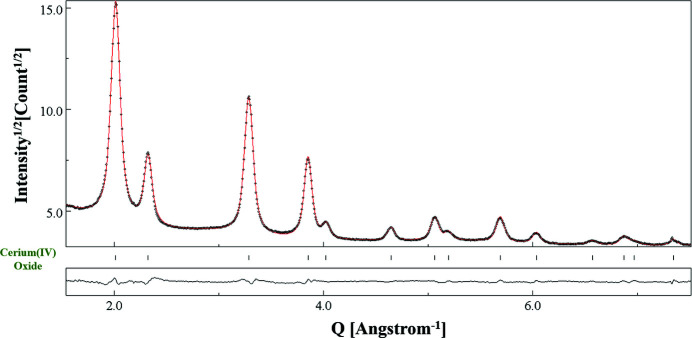
Pattern-matching (Le Bail) fitting of the EPD collected using the SA aperture 40 µm–CL 1080 mm combination. Reliability factors are relatively higher for the EPD analyses for the 40 µm SA aperture due to the limited number of selected scattering domains. *Y* axis: sqrt(intensity); *X* axis: *Q* (Å)^−1^.

The camera lengths estimated on the basis of the 12 diffraction patterns acquired under different conditions are listed in Table 2 ▸. There is not much variation in the calibrated camera lengths upon changing the size of the selected area aperture, as also shown in Fig. 7 ▸. This was expected and confirms that the camera length does not depend on the size of the SA aperture used, although each time the SA aperture is changed, a focus compensation of the electron diffraction using C2 is required. The maximum variation amongst the calibrated values was obtained in the case of the largest camera length, *i.e.* 1360 mm, whereas for the smallest camera length of 658 mm, the deviation was the least. As indicated in Table 2 ▸, we have used Rwp_no_bkg_ as a reliability factor to assess the achievement of a satisfactory fit. In our opinion, this factor is recommended for ED analyses of data sets featuring significant background contributions. We note also that for the 40 µm SA aperture Rwp_no_bkg_ has higher values. This is because the ED data collected using 40 µm SA aperture had the worst statistics, due to the lower number of selected scattering domains [see Fig. 1 ▸(*c*)]. For the particular case of the SA aperture 40 µm–CL 1080 mm combination, the profile fitting of the EPD is shown in Fig. 8 ▸.

### 4.2. Instrumental broadening function

The instrumental function parameters were evaluated using the Le Bail method (Le Bail, 1989 ▸), *i.e.* pattern matching, as this method most accurately converged to the average value of the crystallite size, *i.e.* the quoted 128 Å estimated using the BF images. The profile width (HWHM, ω) and shape, corresponding to the Gaussian fraction (η) of the pseudo-Voigt function, were recorded for all 12 patterns. In particular, the trend of the FWHM data (2ω) was modelled by refining the *U*, *V* and *W* parameters of the Caglioti function (Caglioti *et al.*, 1958 ▸) using the following equation:

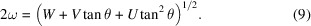

For a pseudo-Voigt function, the relation between FWHM, 2ω, and integral breadth, β, is given by the following equation (de Keijser *et al.*, 1983 ▸):

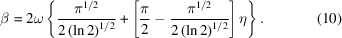

The case of ‘flat top super-Lorentzian’ shape (Wertheim *et al.*, 1974 ▸), *i.e.* η > 1, was not permitted and the maximum value of η was set to 1. Using equation (10), the instrumental broadening based on the integral breadth β was also determined for different EPD data. It might be more suitable than HWHM, particularly for EPD profiles having broadened peaks (Scardi *et al.*, 2004 ▸). Moreover, error due to the wrong estimation of the background level is minimized by the profile fitting.

Table 3 ▸ summarizes the instrumental profile functions calculated for the 12 diffraction patterns. The refinement steps were terminated once there was no significant improvement in the Rwp_no_bkg_ value. A word of caution here: the values of *U*, *V* and W must yield a positive value of 2ω. Once the Caglioti equation parameters *U*, *V* and *W* have been determined at the end of the refinement loop, equation (9) is a function of a single variable: θ. Instead of determining the FWHM at a single value of θ, we calculated the FWHM at θ = 0, 0.25, 0.5, 0.75 and 1° (corresponding to *Q* values from 0 to 8), values interesting for electron diffraction patterns of real materials. Thereafter, an average value of FWHM was determined for each case, listed in Table 4 ▸.

**Figure 9:**
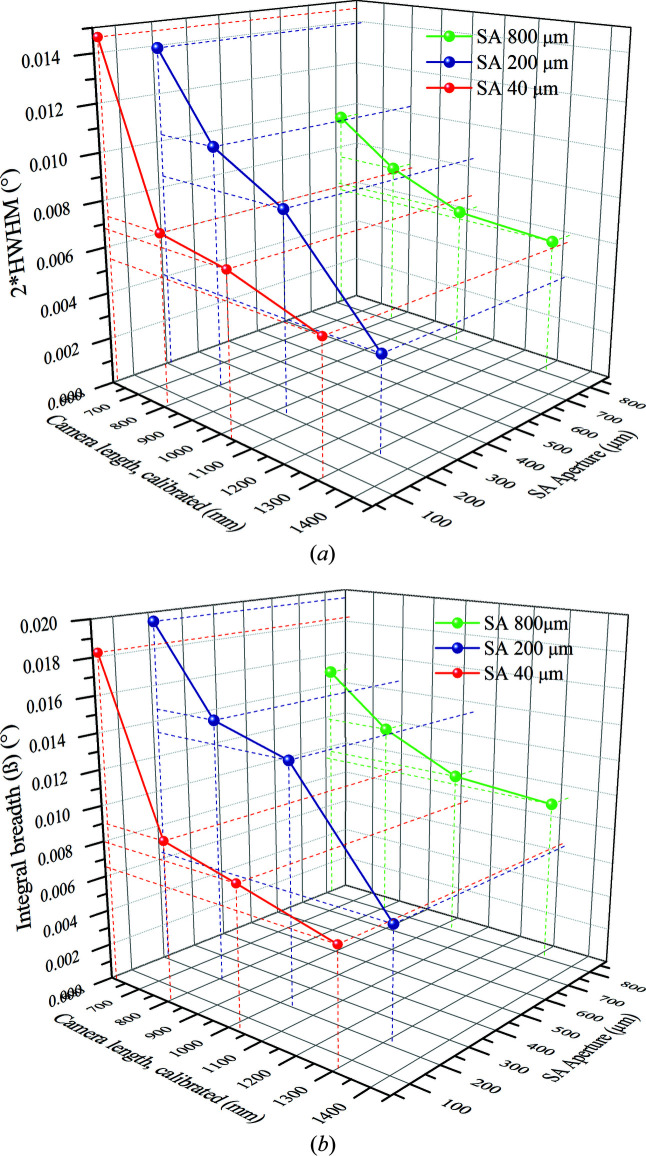
3D plots showing the trend of FWHM values (*a*) and integral breadth (*b*) for different SA aperture–CL combinations.

Interestingly, for a particular SA aperture, the FWHM varies with the CL. In general, for the smallest CL, *i.e.* 658 mm, we have the highest value of FWHM: 0.01468°. On the other hand, for the largest CL, *i.e.* 1360 mm, we have the smallest value of 0.00412°. This is most likely related to the large detector broadening, also reported by Zuo *et al.* (2019 ▸), at shorter camera lengths. Larger camera lengths of 1360 and 1080 mm have closer FWHM values, just like the shorter camera lengths of 844 and 658 mm. Overall, it is safe to assume that instrumental broadening function calibration must be performed for different SA apertures and CLs. The discrepancy in the broadening value is expected as changing the size of the aperture and camera length requires adjustments in the overall lens conditions, particularly C2 current intensity for a change in the SA aperture. The trend is shown in Fig. 9 ▸(*a*) with the help of a 3D plot, having FWHM, calibrated camera length and SA aperture on the three axes. A similar trend was obtained for β values, listed in Table 5 ▸. These have been graphically represented in Fig. 9 ▸(*b*), with β plotted along the vertical axis instead of FWHM. These corresponding magnitudes of FWHM/β for different SA aperture–CL combinations may be used while performing the profile fitting analysis of real samples having large anisotropy or texture.

### 4.3. Two-stage calibration: microstructure determination from EPD

Table 6 ▸ for 800 µm aperture, Table 7 ▸ for 200 µm aperture and Table 8 ▸ for 40 µm aperture illustrate the refined EPD data: lattice parameters, average crystallite sizes and reliability parameters. In these tables, ‘PM’ refers to pattern matching (Le Bail decomposition); ‘kinematical’ indicates that the kinematical approximation is considered for structure factor determination; and ‘Blackman’ indicates the two-beam dynamic correction.

**Figure 10:**
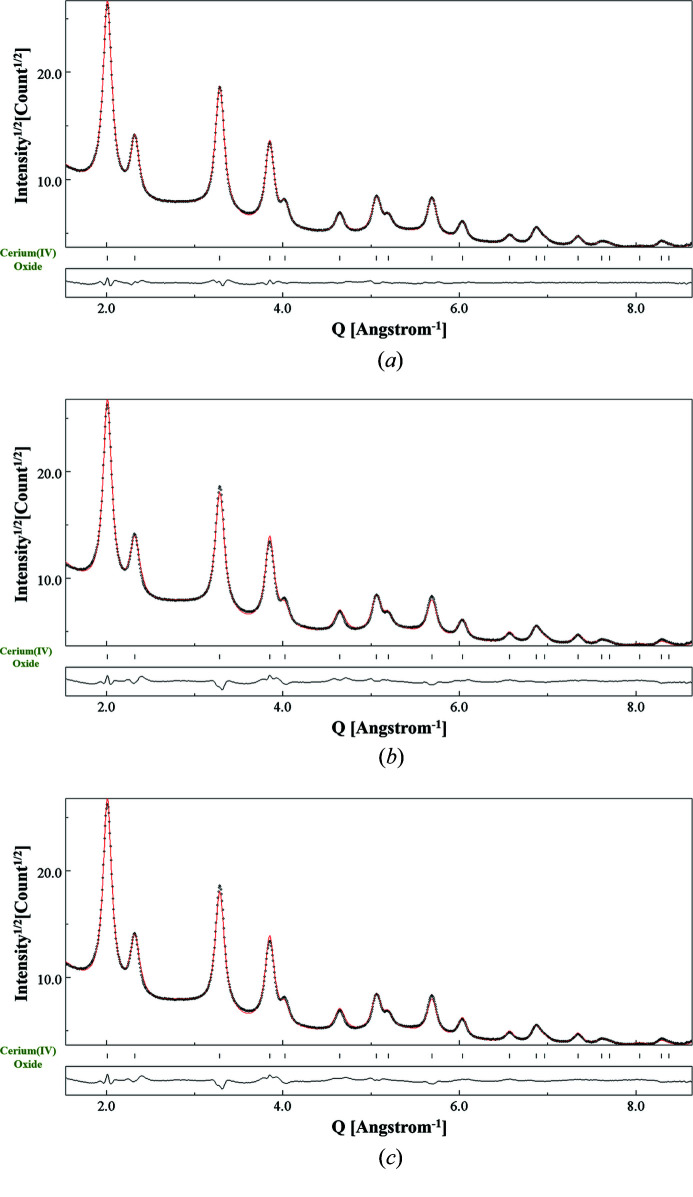
Results of the combined analysis of CeO_2_ nanopowders for EPD patterns treated (*a*) using a pattern-matching mode (Le Bail), (*b*) using the kinematical approximation and (*c*) using the kinematical approximation with the Blackman two-beam dynamic correction. The data refer to the SA aperture 200 µm–CL 1080 mm combination. *Y* axis: sqrt(intensity); *X* axis: *Q* (Å)^−1^.

For a selected case of 200 µm SA aperture and 1080 mm CL, Fig. 10 ▸ shows the EPD profile fitting for the three cases: Le Bail method [Fig. 10 ▸(*a*)], kinematical approximation [Fig. 10 ▸(*b*)] and Blackman two-beam correction [Fig. 10 ▸(*c*)]. For all SA aperture–CL combinations, the crystallite size(s) obtained through the pattern matching mode was found to be closer to the value ‘certified’ by XRD, as highlighted in Tables 9 ▸–11 ▸ ▸. Crystallite sizes along two crystallographic directions – [111] and [100] [reported throughout corresponding to the (200) planes] – have also been listed. Lower reliability parameters have been obtained with the Le Bail method. It was evident that the Le Bail method worked efficiently in fitting the individual intensities. This is the reason why the crystallite sizes along the two crystallographic directions show the least deviation from the average value using the Le Bail method (see Tables 9 ▸–11 ▸ ▸). We did not observe much of a variance in the results when the Blackman two-beam correction was implemented. This is evident from the microstructural features in Tables 6 ▸–11 ▸ ▸ ▸ ▸ ▸ obtained from profile fitting shown in Figs. 10 ▸(*b*)–10 ▸(*c*) and other SA aperture–CL combinations.

**Figure 11:**
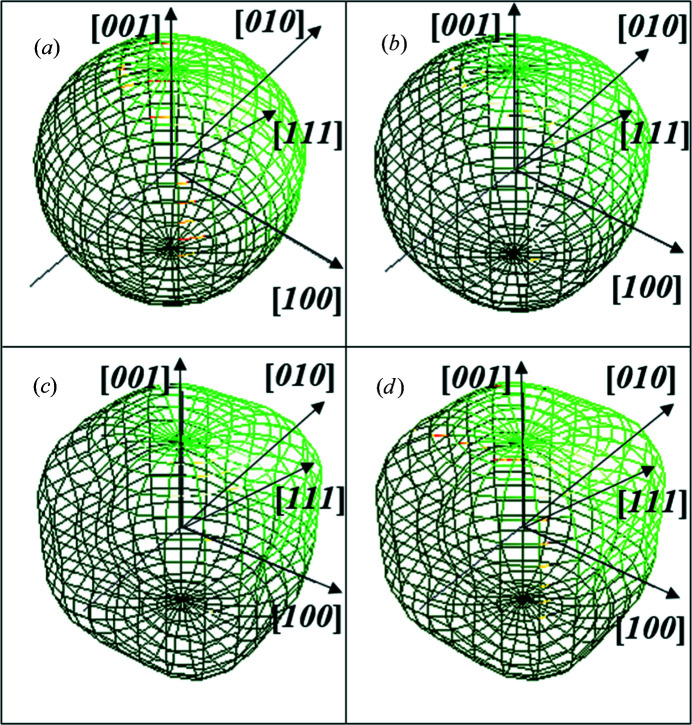
Crystallite shapes modelled on the basis of (*a*) XRPD data showing ellipsoidal geometry and (*b*) ED data following the Le Bail method depicting similar geometry to that in (*a*); (*c*), (*d*) ED data under kinematical and dynamical two-wave approximations, respectively, displaying an irregular pseudo-cubic shape. (*b*)–(*d*) refer to the SA aperture 200 µm–CL 1080 mm combination.

Once we have accounted for *g*(*x*), the deviation of *h*(*x*) from *g*(*x*) can be associated with *f*(*x*), *i.e.* the sample microstructure. The increment in line broadening is thereafter due to two sample characteristics: the finite size of the crystallites and the r.m.s. microstrains (de Keijser *et al.*, 1983 ▸). This latter, *i.e.* the (*hkl*) dependence of the r.m.s. microstrains, can be attributed to defects – for instance, dislocations – present in our standard material. Using Popa rules, including two harmonic coefficients Ro and R1, it is possible to obtain a relatively good estimation of the crystallite shape. Only the Ro and R1 coefficients from equation (8) were considered for crystallite shape 〈*R*_*hkl*_〉 decomposition in both XRD and ED data refinements, although the crystallite shape obtained from these different sets of data turned out to be different. As can be seen in Fig. 11 ▸(*a*), XRD data refinement based on Popa rules yields a rather ellipsoidal shape, which would have been spherical if the crystallites displayed no anisotropy. Along the [111] direction, the crystallites have a dimension of 128 Å, while along the [100] direction, the crystallites have a dimension of 119 Å. ED data refinement based on the Le Bail method generated similar shapes and sizes: 129 Å along the [111] direction and 108 Å along the [100] direction, as shown in Fig. 11 ▸(*b*). However, a pseudo-cubic shape was obtained both for the kinematical approach and when dynamical two-beam correction was considered, shown in Figs. 11 ▸(*c*) and 11 ▸(*d*).

As far as reliability of the lattice parameters, crystallite size and shape is concerned, preference should be given to XRD-based results (Boullay *et al.*, 2014 ▸). Rietveld refinement of XRD data collected from the bulk CeO_2_ sample has superior chances of approaching the true ‘average’ crystallite size and shape, considering the much larger volume sample involved in the analysis.

Still, we expected the dynamical approach to converge towards the true sample microstructural features, as demonstrated by Boullay *et al.* (2014 ▸). The consequences in this scenario are twofold. Firstly, without any prejudice, the kinematical approach has been successfully implemented and found to be satisfactory, as also reported in other studies (Kim *et al.*, 2009 ▸; Weirich *et al.*, 2002 ▸). Secondly, although dynamical two-beam correction is crucial in the ED analyses, dynamical scattering is strongly dependent on the state of aggregates and the local thickness of the agglomerates. A small thickness can attenuate the dynamical scattering, rendering the kinematical approach equally reliable and preferable. The calculation based on the dynamical theory will produce the same results as the kinematical approach, if the analysed region of the sample is thin enough (Li, 2010 ▸). Moreover, the lack of any preferred orientation also has an overall effect on the reduction of dynamical scattering (Zuo *et al.*, 2019 ▸). These are the probable causes for the similar results that have been given in Tables 6 ▸–11 ▸ ▸ ▸ ▸ ▸. Nevertheless, the inclusion of the dynamical two-beam correction in the *MAUD* software is ingenious, and it will be interesting to use this approach with samples having crystals that are too thick for the kinematical approach to provide reliable results.

## 5. Conclusions and perspectives

In this paper, we demonstrated the applicability of Rietveld refinement to the analysis of electron diffraction ring patterns, as concerns the methodology for calibrating camera lengths and instrumental broadening based on FWHM and integral breadth. The approach has been implemented using a CeO_2_ nanocrystalline powder sample, taken as a standard. XRD tests were conducted on the same sample to evaluate the reference values for the microstructural features, like crystallite size and r.m.s microstrain, both contributing to the broadening of the diffracted lines. The Le Bail method was able to match the experimental and calculated data better than all the other considered models. Hence, it can be regarded as best suited for calculating the sample features, as determined from the full-pattern fitting procedure and direct observation.

As expected, the instrumental broadening function was found to depend on the size of the selected area aperture and on the camera length used to collect the diffraction patterns. It varied from 0.01468 to 0.00412° based on FWHM, and from 0.01955 to 0.00647° based on integral breadth. For the calibration of the camera length, the calibrated values were found to be close to the nominal instrumental values.

To verify our hypothesis, keeping the instrumental broadening function fixed, we calculated the microstructural parameters using the complete set of 12 CeO_2_ diffraction patterns deriving from the combination of different experimental parameters. For the standard CeO_2_ sample, the kinematical approximation yielded results identical to those obtained using the dynamical two-wave approximation approach. This is primarily due to the lack of any preferred orientation, and also the nanometric size and, thereby, the thickness of the particle.

This developed approach turns out to be an effective tool for determining the instrumental broadening parameters, necessary to apply the proposed methodology to specimens having multiple phases, featuring even broad distributions of crystallite size. These aspects will be presented in Part II of this series of papers.

## Supplementary Material

This file contains the step by step procedure to carry out the proposed analysis in Materials analysis using diffraction (MAUD) software. DOI: 10.1107/S1600576722006367/nb5328sup1.pdf

## Figures and Tables

**Table 1:** XRPD profile fitting: reliability factors, cell parameter and crystallite size refinements obtained from the analyses of CeO_2_ nanopowder

Method	*R*_wp_ (%)	*R*_Bragg_ (%)	*a* (Å)	Average crystallite size (Å)
XRD	13.41	8.79	5.4101 (3)	124.9 (4)

**Table 2:** Calibrated camera lengths for different SA apertures (800, 200 and 40 µm) and the reliability factor, Rwp_no_bkg_, for each profile fit

	SA 800 µm		SA 200 µm		SA 40 µm	
Camera length, instrument (mm)	Camera length, corrected (mm)	Rwp_no_bkg_ (%)	Camera length, corrected (mm)	Rwp_no_bkg_ (%)	Camera length, corrected (mm)	Rwp_no_bkg_ (%)
1360	1322.58 (1)	5.05	1324.84 (6)	6.01	1315.34 (3)	13.39
1080	1039.46 (1)	5.70	1036.69 (1)	6.96	1034.96 (3)	13.36
844	812.249 (8)	5.40	808.15 (1)	6.53	811.40 (6)	13.55
658	618.874 (7)	6.11	617.21 (2)	7.23	618.37 (6)	13.08

**Table 3:** Caglioti function parameters (*W* and *V*) and Gaussian fraction (η) determined for different camera length–SA aperture combinations The parameter *U* is zero for all camera length–SA aperture combinations and hence not tabulated.

	SA aperture 800 µm			SA aperture 200 µm			SA aperture 40 µm				
Camera length, instrument (mm)	*W*	*V*	η	*W*	*V*	η	*W*	*V*			η
1360	1.71 (3) × 10^−5^	0.00224 (6)	1	3.05 (4) × 10^−5^	−0.00141 (7)	1	7.30 (3) × 10^−5^	−0.00434 (3)			0.221 (3)
1080	6.44 (5) × 10^−6^	0.00435 (1)	1	6.050 (2) × 10^−5^	0.00120 (4)	1	9.24 (1) × 10^−5^	−0.0046 (1)			0.208 (5)
844	2.56 (2) × 10^−5^	0.00399 (2)	1	7.6 (1) × 10^−5^	0.00363 (4)	0.713 (4)	1.207 (1) × 10^−4^	−0.0065 (2)			0.317 (5)
658	4.8 (1) × 10^−5^	0.0051 (1)	1	1.331 (5) × 10^−4^	0.00736 (7)	0.664 (3)	2.70 (2) × 10^−4^	−0.0061 (3)			0.362 (3)

**Table 4:** FWHM, 2ω, determined for different camera length–SA aperture combinations

	FWHM: 2ω (°)		
Camera length, instrument (mm)	SA 800 µm	SA 200 µm	SA 40 µm
1360	0.00594	0.00412	0.00558
1080	0.00626	0.00857	0.00683
844	0.00759	0.01035	0.00739
658	0.00955	0.01396	0.01468

**Table 5:** Integral breadth, β, determined for different camera length–SA aperture combinations

	Integral breadth: β (°)		
Camera length, instrument (mm)	SA 800 µm	SA 200 µm	SA 40 µm
1360	0.00933	0.00647	0.00656
1080	0.00983	0.01347	0.00799
844	0.01194	0.01477	0.00906
658	0.01499	0.01955	0.01831

**Table 6:** Microstructural parameters and Rwp_no_bkg_ refinements resulting from the size and shape analyses of CeO_2_ nanopowders for different camera lengths with 800 µm SA aperture

	EPD PM			EPD kinematical			EPD Blackman			
Camera length (mm)/SA aperture: 800 µm	*a* (Å)	Average crystallite size (Å)	Rwp_no_bkg_ (%)	*a* (Å)	Average crystallite size (Å)	Rwp_no_bkg_ (%)	*a* (Å)	Average crystallite size (Å)	Rwp_no_bkg_ (%)	
1360	5.41025 (6)	112.36 (6)	5.07	5.41024 (9)	110.8 (1)	7.78	5.41022 (9)	111.6 (1)	7.79	
1080	5.41000 (5)	112.64 (8)	5.02	5.41015 (9)	107.8 (1)	8.22	5.40999 (9)	108.8 (1)	8.15	
844	5.41014 (6)	119.83 (7)	5.25	5.4102 (1)	114.0 (1)	9.32	5.4102 (1)	115.02 (9)	9.21	
658	5.4116 (8)	121.0 (1)	5.99	5.4115 (1)	118.3 (2)	8.87	5.4115 (1)	118.6 (2)	8.89	

**Table 7:** Microstructural parameters and Rwp_no_bkg_ refinements resulting from the size and shape analyses of CeO_2_ nanopowders for different camera lengths with 200 µm SA aperture

	EPD PM			EPD kinematical			EPD Blackman			
Camera length (mm)/SA aperture: 200 µm	*a* (Å)	Average crystallite size (Å)	Rwp_no_bkg_ (%)	*a* (Å)	Average crystallite size (Å)	Rwp_no_bkg_ (%)	*a* (Å)	Average crystallite size (Å)	Rwp_no_bkg_ (%)	
1360	5.4103 (2)	114.63 (7)	5.80	5.4110 (4)	110.01 (8)	9.79	5.4110 (4)	109.79 (8)	9.51	
1080	5.41022 (8)	121.3 (1)	6.96	5.4102 (1)	115.22 (6)	9.60	5.4102 (1)	115.65 (7)	9.55	
844	5.4102 (1)	118.5 (1)	7.45	5.4100 (1)	112.5 (2)	9.86	5.4101 (1)	111.34 (7)	9.69	
658	5.4108 (2)	121.18 (6)	7.34	5.4110 (2)	114.8 (1)	10.77	5.4108 (2)	115.0 (1)	10.81	

**Table 8:** Microstructural parameters and Rwp_no_bkg_ refinements resulting from the size and shape analyses of CeO_2_ nanopowders for different camera lengths with 40 µm SA aperture

	EPD PM			EPD kinematical			EPD Blackman					
Camera length (mm)/SA aperture: 40 µm	*a* (Å)	Average crystallite size (Å)	Rwp_no_bkg_ (%)	*a* (Å)	Average crystallite size (Å)	Rwp_no_bkg_ (%)	*a* (Å)			Average crystallite size (Å)	Rwp_no_bkg_ (%)	
1360	5.4102 (1)	121.5 (2)	13.51	5.4107 (2)	114.9 (4)	20.67	5.4106 (2)			111.2 (3)	20.45	
1080	5.4102 (1)	123.0 (2)	13.40	5.4106 (2)	120.0 (3)	20.36	5.4106 (2)			118.0 (2)	20.24	
844	5.4115 (4)	120.1 (2)	13.93	5.4130 (7)	110.9 (2)	22.57	5.4131 (7)			115.9 (4)	22.68	
658	5.4117 (4)	113.6 (3)	12.77	5.4120 (8)	108.3 (6)	20.46	5.4116 (8)			107.6 (8)	20.34	

**Table 9:** Crystallite sizes along the directions [111] and [100] and the average value for different CL combinations of SA aperture 800 µm, calculated using pattern matching (Le Bail decomposition), the kinematical approximation and the Blackman two-beam dynamic correction

	EPD PM: crystallite size (Å)			EPD kinematical: crystallite size (Å)			EPD Blackman: crystallite size (Å)		
Camera length (mm)/SA aperture: 800 µm	[111]	Average	[100]	[111]	Average	[100]	[111]	Average	[100]
1360	123.47	112.36 (6)	95.70	125.5	110.8 (1)	88.8	126.1	111.6 (1)	89.9
1080	122.28	112.64 (8)	98.17	122.8	107.8 (1)	85.4	123.7	108.8 (1)	86.7
844	122.61	119.83 (7)	115.64	130.9	114.0 (1)	88.7	130.85	115.02 (9)	91.28
658	135.2	121.0 (1)	99.8	137.4	118.3 (2)	89.8	138.0	118.6 (2)	89.5

**Table 10:** Crystallite sizes along the directions [111] and [100] and the average value for different CL combinations of SA aperture 200 µm, calculated using pattern matching (Le Bail decomposition), the kinematical approximation and the Blackman two-beam dynamic correction

	EPD PM: crystallite size (Å)			EPD kinematical: crystallite size (Å)			EPD Blackman: crystallite size (Å)		
Camera length (mm)/SA aperture: 200 µm	[111]	Average	[100]	[111]	Average	[100]	[111]	Average	[100]
1360	124.10	114.63 (7)	100.42	127.26	110.01 (8)	84.14	126.29	109.79 (8)	85.04
1080	129.6	121.3 (1)	108.9	134.75	115.22 (6)	85.92	134.46	115.65 (7)	87.45
844	133.1	118.5 (1)	96.6	131.2	112.5 (2)	84.5	135.15	111.34 (7)	87.39
658	127.52	121.18 (6)	105.25	130.4	114.8 (5)	91.4	130.7	115.0 (1)	91.5

**Table 11:** Crystallite sizes along the directions [111] and [100] and the average value for different CL combinations of SA aperture 40 µm, calculated using pattern matching (Le Bail decomposition), the kinematical approximation and the Blackman two-beam dynamic correction

	EPD PM: crystallite size (Å)			EPD kinematical: crystallite size (Å)			EPD Blackman: crystallite size (Å)		
Camera length (mm)/SA aperture: 40 µm	[111]	Average	[100]	[111]	Average	[100]	[111]	Average	[100]
1360	127.6	121.5 (2)	112.4	135.1	114.9 (4)	84.8	129.3	111.2 (3)	84.2
1080	127.5	123.0 (2)	116.3	134.0	120.0 (3)	99.0	131.1	118.0 (2)	98.5
844	121.9	120.1 (2)	117.4	126.3	110.9 (2)	87.9	132.6	115.9 (4)	91.9
658	114.8	113.6 (3)	111.9	120.4	108.3 (6)	90.1	117.4	107.6 (8)	88.0
