# Supplementary material for: Electron diffraction characterization of nanocrystalline materials using a Rietveld-based approach. Part I. Methodology
Source: J Appl Crystallogr. 2022 Aug 1;55(Pt 4):953–65. doi: 10.1107/S1600576722006367 (PMC9348886; doi:10.1107/S1600576722006367)
Supplement: Supplementary file 1 [file j-55-00953-sup1.pdf]

JOURNAL OF APPLIED CRYSTALLOGRAPHY

**Volume 55 (2022)**

**Supporting information for article:**

# Electron diffraction characterization of nanocrystalline materials using a Rietveld based approach. Part 1. Methodology

**Ankur Sinha, Mauro Bortolotti, Gloria Ischia, Luca Lutterotti and Stefano Gialanella**

We have demonstrated in this document the step-by-step procedure to perform microstructural analysis of electron powder diffraction patterns using Materials analysis using diffraction (MAUD) software. All the datafiles and MAUD analysis files can be downloaded as a zipped file at http://maud.radiographema.eu/static/tutorial/EPD.zip

The latest version of MAUD can be downloaded from http://maud.radiographema.eu/ and saved to a local directory. In the windows operating system, we have to use MAUD.bat file for running MAUD. It would be a good idea to see video tutorials at: http://www.youtube.com/user/MaudRietveldProgram for some elementary analysis.

When we run MAUD for the first time (batch file), the window shown in Fig. S-1 is opened. On the top left side, we see the “Datafiles”, “Phases” and “Samples” tabs. Above these, in the taskbar, we have “Floppy disk” for saving the analysis (it is a good practice to save the analysis at different stages), “Eye” for editing an object that has been selected (datafiles, phases, etc.), “Light bulb” for selecting different refinable parameters, “Calculator” for computing the diffraction patterns, “Hammer/ Machine” for initiating the refinement cycle.

**S1. Setting up the Instrument:**

MAUD can be used for diffraction data of neutrons, x-rays, electrons. For every dataset, we first need to define the instrument. In our case, we define our TEM instrument as follows:
Datasets → DataFileSet_x→Edit Object (in the menu taskbar) / press “Eye”. In the General tab of the next window, select “Edit” under Instrument. The window shown in Fig. S-2 will open.

- Here, we can give the name of our instrument. In our case, it is Thermofisher Scientific- TALOS F200 S.
- Make sure that we choose “none cal” for the intensity calibration option for the current analysis.

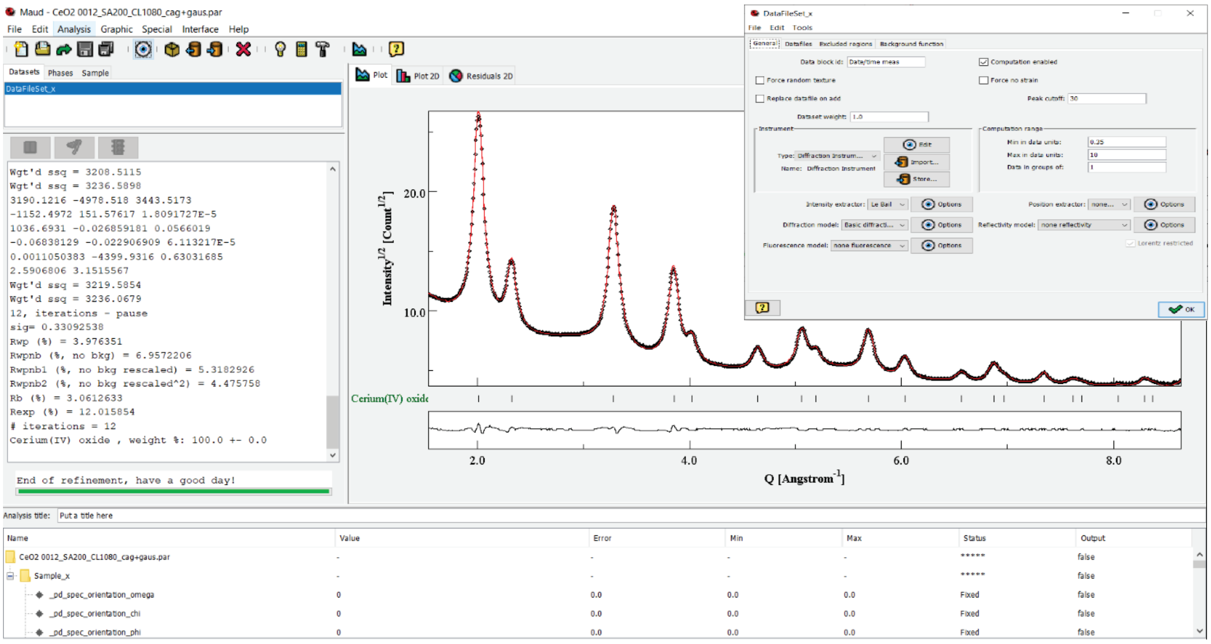


**Figure S1** Main MAUD window on a computer running on Windows 10. When the program is started the plot is blank. Here, data for the CeO2 standard have been entered and refined. The superposed image (top right) shows the window "DataFileSet" with options for modifying the selected Dataset.

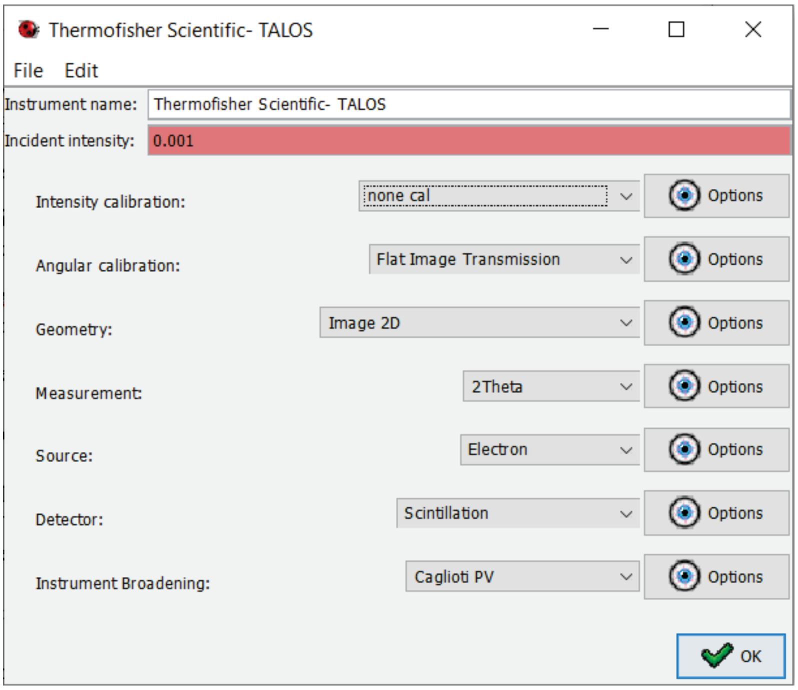


**Figure S2** "Diffraction Instrument" window with general setup for transmission electron diffraction images.

- We can set the value of the incident intensity to 0.001 initially. This value will be refined at a later stage.

- Under the “Angular Calibration” drop-down menu, we have to select “Flat Image Transmission”. Under the options tab, we have to set the detector distance as 1080 mm. This was the camera length as obtained directly from the TEM instrument, which needs to be refined later. The detector distance is an essential parameter for the conversion of coordinates of the 2D image to 2θ values. Later, we can also convert the 2θ values to d-spacing or Q-values. For now, we need not change the remaining parameters, such as center x and center y (in the integration tab) and their errors.
- Under the “Geometry” tab, we choose “Image 2D”.
- For “Measurement”, we select “2Theta”.
- For “Source” we have to select “Electron”. We can click on “Options” and change the default wavelength to 0.0251 (Å), as we had collected the TEM data at 200 kV. The “Dynamical correction (Blackman)” should be checked, while “Crystallite value for thickness” unchecked.
- In the “Instrument Broadening” tab, we should set the default parameters that are compatible with electron diffraction. If we click the “Options” button that is next to the “Caglioti PV” model (Caglioti et al., 1958), we will see the additional tabs. Here, we should remove the asymmetry parameters/or set them to zero. We repeat the same for all the parameters under the “HWHM” and “Gaussianity” tabs. Next, we can close the Instrument editing window.

### S2. Entering 2D image data

- The ImageJ plugin has been embedded in MAUD. Thus, an image in .tif format can be directly opened in MAUD and the resulting data files can be saved as .esg files, which are ASCII files. These contain a list of radial positions or diffraction angles and the experimental intensities corresponding to these. However, if the diffraction data is in a format that is not readily recognized by ImageJ, it needs to be converted to a regular .tif file, preserving the original intensities. To perform the image integration in MAUD using the ImageJ plugin, we proceed as follows: Datasets→ Edit (eye button) → Datafiles → From images (Fig. S-3). A small window showing the typical ImageJ toolbar will be displayed.
- Using the ImageJ menu “File→Open” we can load the diffraction image CeO2 0012_SA200_CL1080.tif. It is advised to work with the raw file, which is already in the 16-bit format and without the magnification scale bar.
- We can set the proper brightness/contrast of the image. From the menu select: “Image→Adjust→Brightness/Contrast”, and press the “Auto” button as many times until we can see clearly also weaker diffraction circles. Thereafter, we can close this window.

- We need to specify the pixel size for the detector. Select “Image→Properties”. A new window will open. Here, change the “Unit of length” to mm and set “Pixel width” and “Pixel height” to 0.014 mm (14 μm/pixel) and press “Ok”. These values refer to the pixel size of the CCD on which diffraction patterns were collected.

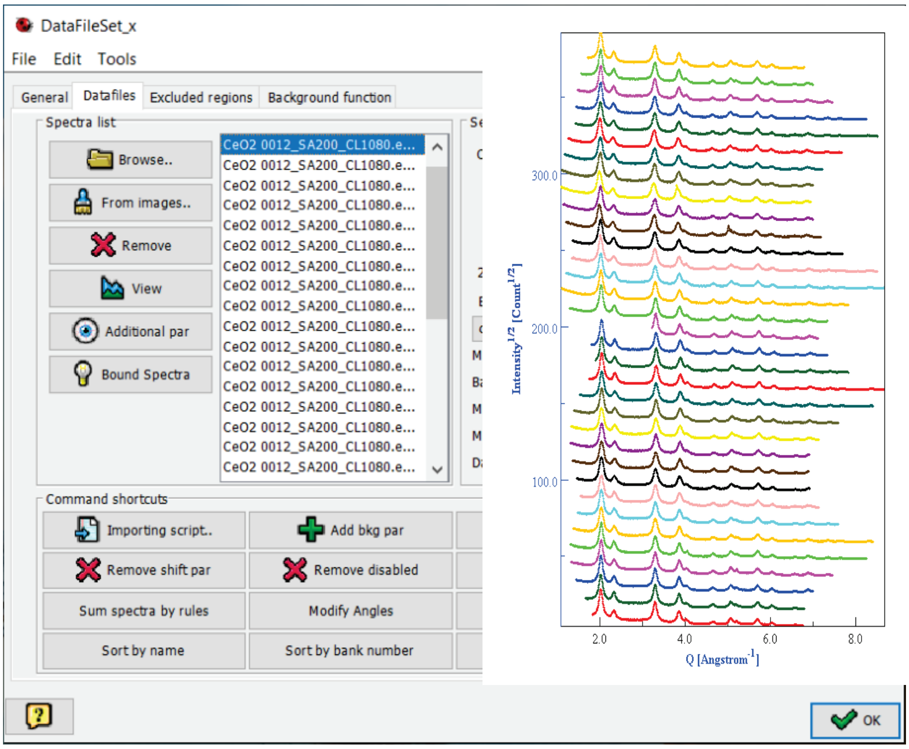


**Figure S3** “Datafiles” in Dataset options window for $CeO_2$ standard. Initially, there is no data in this window. In the view shown here, they have been loaded selecting “From images”. Superposed on the right, we see all the individual diffractograms formed by selecting all the .esg files and pressing “View”.

- The latest version of MAUD facilitates removing the beam stopper shadow that masks a portion of the diffraction rings. For this, select the “Polygon selections” option from the Image J taskbar and select the entire area of the beam stopper. Then, select Process → Math→ Set →Value to -1. Click on Ok. Thus, there will be no contribution from the selected portion of the image. This operation has been shown in Fig. S-4
- Now we proceed to integrate the image by selecting: “Plugins→Maud plugins→Multi spectra from normal transmission/reflection image”. The diffraction image appears as shown in Figure S-5. Also, another window “Choose the integration lines” will appear showing a list of parameters. For this sample, the Sample-Detector distance was 1080 mm. We need to adjust the “Center X (unit: mm)” and “Center Y (unit: mm)” causing the tracker circle (in red) to move towards the center (X = 29.43 and Y = 28.43). We should also adjust the tracker radius so that it coincides with the first ring. Then we update the plot by pressing “Update” and the changes made would be visible. We can set the “Number of Spectra” to 36, i.e. the

image will be integrated with 10° sectors. The angle Omega (ω) should be set to 90°. This will serve to bring the normal to the sample into the pole figure center. We can leave the rest of the entries to 0. However, we should be sure that "Reflection image" and "2-Theta angles calibrated" buttons are unchecked since we are working with a transmission image.

- When we press on "OK", the integration will start and the software will ask the location to save the ASCII files. We can choose the directory ( better not the MAUD directory) and give a name such as CeO2 0012_SA200_CL1080.esg.

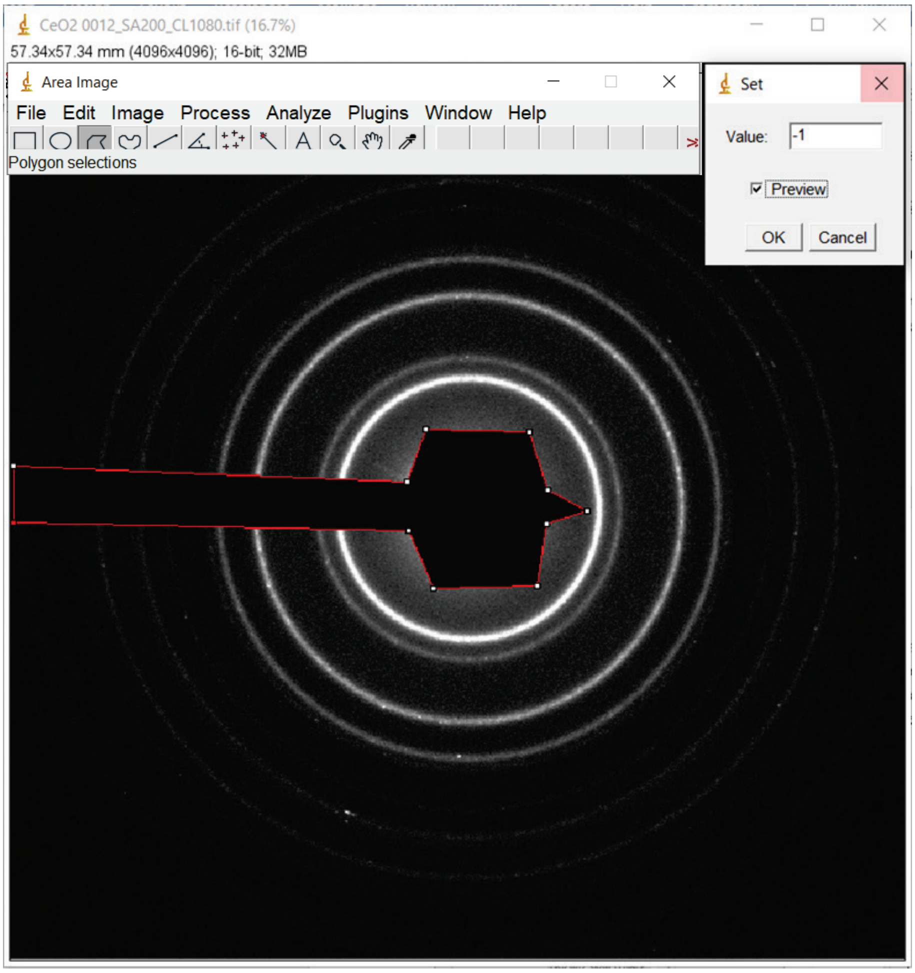


**Figure S4** The area of the diffraction pattern of $CeO_2$ masked by the beam stopper is selected (red boundary lines) using "Polygon selections" from the ImageJ menu window shown on the top left. The intensity values for the selected region are set to -1, shown on the top right.

- At this point, we can close the diffraction image and ImageJ windows. In the MAUD dataset editing window, the .esg files would now be listed in the "Spectra list" panel, shown in Fig. S-3. We can see all the individual diffractograms by selecting all of them (Ctrl+A) and then clicking "View". However, first, we should delete the diffractograms numbers 17 and 18 (note that the numbering of diffractograms starts from 0), falling in the region of the beam stopper. Thus, 34 individual diffractograms shown in Fig. S-3 can be obtained. If we now

close the dataset window, a summation of all the patterns would be visible in the main MAUD window plot panel (Figure S-1).

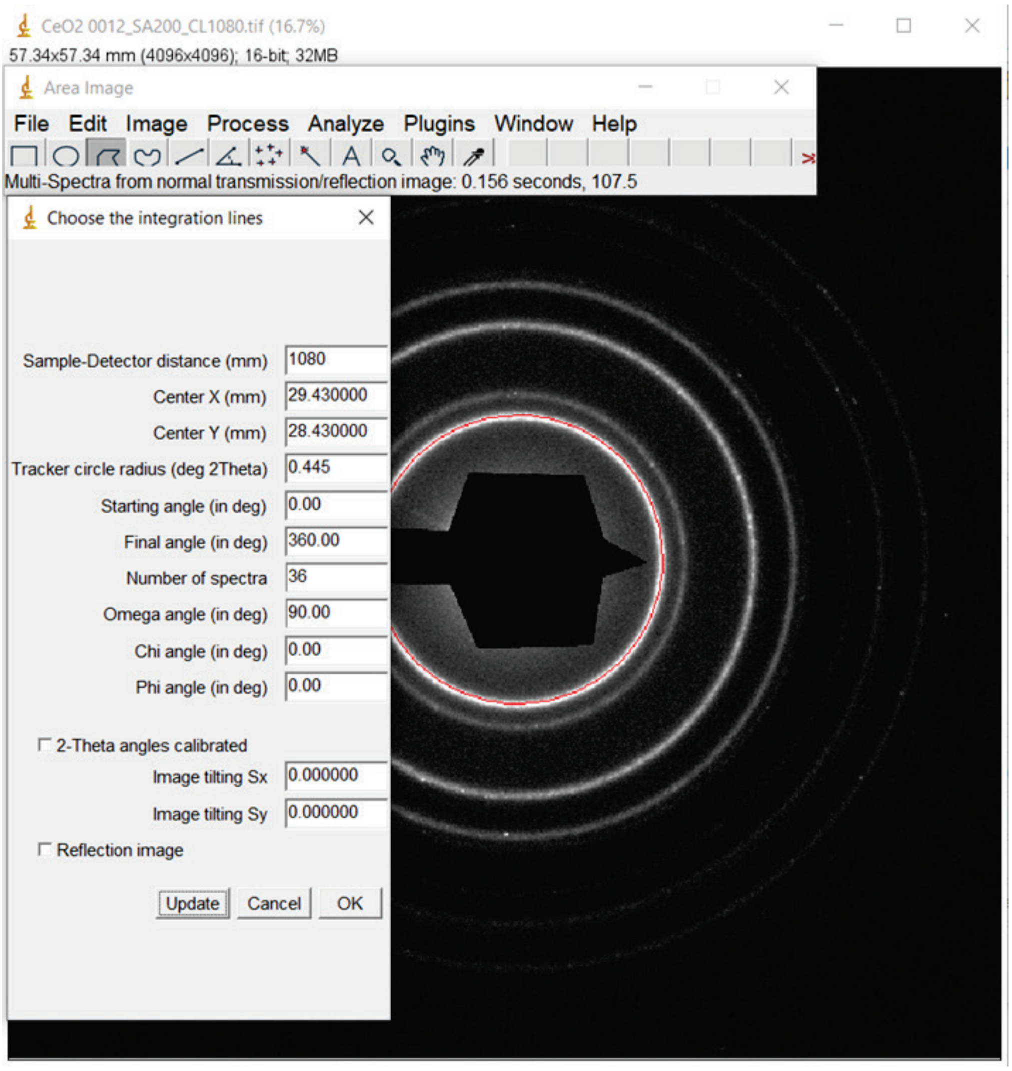


**Figure S5** ImageJ setup for $CeO_2$ diffraction image processing to generate a set of patterns by azimuthal integration of the 2D image. The red circle is used for aligning the image in the center.

### S3. Refinement range and background

We can restrict the range for the refinement. This can be done by selecting the "General" tab of the dataset editing window (Fig. S-1, inset) and changing "Min in data units" and "Max in data units" values. Units for those are the same as the actual diffraction data. Thus, $2\theta$ in degrees for this case. Next, we should set the background. For selecting the background model, switch to "Background function" tab following: Datasets → DataFileSet_x → Eye button → Background function. By default, a polynomial background is used in MAUD. We need to add 5 parameters to approximate the background by a 4$^{th}$ order polynomial. We have to click "Add parameter" or "Remove parameter" buttons to set the number of parameters to 5.

We also need to account for the sharp increase in the background at low 2θ/ Q values. At the same time, the contribution of the carbon film needs to be accounted for. We proceed as follows: Background function tab → Background Peaks → add term. We have to add three terms, one corresponding to the direct beam, and two for the Halos from the carbon film. For the direct beam, we can give initial values as follows- Height: 10; position: 0; HWHM: 0.5; Eta:1. We can set to refine these parameters, except for the position value.

Initial values for the Halos 1: Height: 1; position: 0.6666; HWHM: 0.0889; Eta: 0.3699.

Initial values for the Halos 2: Height: 1; position: 1.2124; HWHM: 0.1053; Eta: 0.5878.

We can refine the Height parameter but others should be kept fixed.

Please refer to Section S8, wherein we have demonstrated the procedure to obtain these values.

### S4. Phase and initial parameters adjustment

The "Phases" tab in the main MAUD window (S-1) contains the crystallographic and microstructural data of all the phases present in the sample. These are lattice parameters, space group, atomic positions, occupancy, crystallite size, microstrains, texture, etc. The basic sample features can be entered through "crystallographic information files" (.cif). These files can be downloaded from different databases, such as the ICSD (ICDD) or the COD (http://www.crystallography.net). Some examples of phases are also available in the MAUD file "structures.mdb".To import a structure from a .cif file we have to press the "Cylinder with arrow out" button on the taskbar or select the "Edit→Load object from CIF…" menu. However, in our case, we need to import sample characteristics from the profile fitting of $CeO_2$ XRD data. So, we proceed as follows: Phases → Load object from CIF→XRD file (CeO2_00_XRD_cal.par) → Cerium oxide →select and choose. Following these steps, all structural and microstructural parameters obtained from the XRPD analysis would be taken as input for the EPD analysis. We have to be careful and not load the $CeO_2$.cif file downloaded from the COD.
After entering all the above information, pressing the "Calculator" button on the main page (Figure S-1) shows diffraction patterns based on the instrumental and phase parameters provided by the user and compares them with the experimental data. This can be viewed with the "Plot" tab (selected by default) above the pattern in MAUD main window. Here, an average of all patterns from a dataset is shown. Otherwise, with the "Plot 2D" tab a stack of all individual 34 patterns can be displayed. In the 2D representation, model patterns are at the top and experimental patterns are at the bottom.

For diffraction data, we can choose different scales for representing the intensity values. The square root scale intensity representation is useful, as smaller peaks could also be visible. This can be selected or changed in the "Graphic→Plot options" menu.

The calculated intensity values are different from the experimental values. So, we should adjust the scale factor (beam intensity in MAUD). This can be performed "live" by selecting the parameter list

below the plot, i.e., the lowermost portion of the main MAUD window (Figure S-1). In the parameter list, we should enlarge the tree-table and scroll to reach the parameter "_pd_proc_intensity_incident". It is present in the instrument in the dataset. We can make the "Value" column of the tree- table sufficiently large by changing its border length. We have to click on the value of the entity that we want to edit (one click). Then, in the text field, we can assign the value by which we want to increase or decrease the selected entity. The change in the value is performed by pressing the corresponding arrows. The changes hence made would be directly visible in the plot. We can set the initial value to 2.052E-5. After making the suitable adjustments, we should exit the editing mode by clicking anywhere in the tree table, except on a value.

We would notice disagreement between the peak positions of the experimental and calculated profiles. This is because the camera length of 1080 mm that we entered before is not the calibrated value. To approach the calibrated value, proceed as follows in the tree table: Thermofischer Scientific- Talos→ Flat Image Transmission→pd_instr_dist_spec/detc. We can bring this value down to 1039 mm. Also, we set it to refined.

At this stage, we can set to refined background parameters "_riet_par_background_pol0 *to* _pol4". Then we go ahead with one refinement cycle by pressing the hammer/machine icon. The least-square minimization will start, visible on the left side of the main MAUD window. We can increase the number of cycles by using the "slider". Usually, 5-7 refinements should be sufficient. If the refinement cycle is successful, we will notice that the experimental and calculated intensities show less difference than before.

### S5. Refine instrument parameters

In the next refinement cycle, we account for the centering and tilting errors. Under the "Flat Image Transmission" option, set all the remaining parameters to be refined, except for "inst_ang_calibration_ratio_pixels". Then we go for the next refinement cycle by pressing the "Hammer/Machine" icon. This will adjust the reflection positions. We should save the analysis as a MAUD parameter file in the .par format. (e.g. name it CeO2 0012Ceta_ SA200_CL1080_02_ cent+tilt.par) . We should proceed using the menu item: "File→Save analysis as…". This parameter file contains all essential information about the sample features, phases, and instrument. It can be used as the starting analysis file for other diffraction images.

Finally, we proceed to refine the instrument broadening parameters (W, V, and U) in the "Caglioti PV" model. In MAUD, the designation is as follows: W= _riet_par_caglioti_value0; V=_riet_par_caglioti_value1; U=_riet_par_caglioti_value2. Caglioti parameters essentially describe instrument broadening, with respect to peak width, shape, and asymmetry. These parameters also explain the Gaussian plus Lorentzian mixing (Caglioti *et al.*, 1958). These instrument peak shape and

width parameters are essential if we want to study the line broadening analysis of real samples to determine their microstructure.

Caglioti parameters are very sensitive to changes in their values and a strict order needs to be followed to obtain the desired results. We describe the procedure for transmission electron image data where the $2\theta$ range is limited.

It is advisable to start with non-zero values. So, set the first FWHM parameter "_riet_par_caglioti_value0" to 1E-5, manually by using the parameter tree list at the bottom of the main window. We can set the second FWHM parameter to 0.001. Set both these parameters to be refined. There should be no need to use the third parameter, as we are not dealing with a large 2θ range. If we want to see an enlarged view of any particular peak, we should select an area around that peak by pressing the "right" click and dragging. Then, to zoom out and see the full pattern view, we have to double click on the plot. Otherwise, we can also right-click on the plot and select "reset scale". Next, we go for a refinement cycle.

If we look at the plot window, we would notice that the peak intensities fit rather poorly. So, we can do an intensity-independent Le-Bail fitting for each pattern. We proceed as follows: Cerium oxide→edit (eye button) → Advanced models → Texture → choose arbitrary texture. Click on Ok.

Next, we can fix all the parameters related to the intensity or scale factors, as these are not needed with the Le-Bail fitting. So, set "_pd_processing_intensity_incident" as fixed. Perform another refinement cycle.

Next, we can refine the first gaussian term "_riet_par_gaussian_value0". It is advisable to start with a non-zero value. So, manually set the value to 0.1 before going for the refinement cycle. Once the refinement cycle is completed, check the refined value of this term. If the value goes above 1, we will encounter an error. In this case, manually set the value to 1 and put the status as fixed.

On some occasions after a refinement cycle, we may encounter an error as "Cholesky negative diagonal" on the MAUD output panel. This means that some of the parameters that the software was trying to refine were ill-conditioned to be refined with the given set of conditions. Such parameters can be viewed in the MAUD output file (with the .lst extension). Alternatively, such refined parameters would have the value "-1" in the "Error" column of the tree structure. We may rectify the problem by putting suitable correct values for such parameters, or altogether, refraining from refining them.

### S6. Saving the results

At the last refinement stage, the calculated peaks should fit the experimental peaks adequately. This should be checked both in the "Plot" as well as "Plot2D" displays. We must save this file. To export this instrument setting for use in the refinement cycle of other samples, proceed as follows: DataFileSet_x→ Edit(eye button) →General tab →Store button, in the "Instrument" panel. Following

these steps, we can save the instrument settings for a particular SA aperture-CL combination as a separate file in our directory (e.g., with name, Thermofischer_Talos_SA200_CL1080.mdb).

Using the $CeO_2$ standard, we have now obtained the refined values of relevant instrument parameters. While applying them to diffraction patterns from other samples measured under identical conditions, we should set all Caglioti parameters as "fixed". We should also set the camera length as "fixed", as using the standard $CeO_2$ material, it has now been calibrated.

### S7. Two-stage calibration: Microstructural parameters of $CeO_2$ from EPD

We should start the analysis again with a blank MAUD window, so start by double-clicking on the .bat file. We need to load the instrument file that we had saved at the last step in section S6. We proceed as follows: Data_File_Set_x→ Edit(eye button) →General tab → Import → $CeO_2$ _Calibration.mdb → Choose instrument→Ok

In this file, we should make sure that camera length and Caglioti parameters have been set to "Fixed". We do not need to import the electron diffraction image again as we already have the ASCII files in the .esg format. So we can directly proceed as follows: Data_File_Set_x → Edit(eye button) →Datafiles tab → Browse and locate the CeO2 0012_SA200_CL1080.esg file previously stored. Another way to load the .esg file is to drag and drop it in the blank region in the Data_File_Set_x window. We can remove the diffractograms number 17 and 18. Then click on "OK" to obtain the 1D plot in the MAUD main window.

Next, we have to load the phase. This time we will not be importing the phase information from the XRD analysis, as we want to determine the microstructure using EPD. So, we switch to the "Phases" tab and drag and drop the CeO2.cif file in the blank space just below it. Alternatively, we can load this file following the procedure explained in section S5. Press on the "Calculator" icon and we will see the calculated and experimental data together.

Here we should proceed with the setting of the correct background and detector tilting and centering as explained previously. We should also add the contribution of the carbon film towards the background. Ideally, we should not need to change the errors due to the detector tilting and image center, and these should be taken from the calibration file and fixed. It will be a good idea to save the analysis at this stage. We may save as CeO2 0012 Ceta_SA200_CL1080_04_two_stage.par. Once these steps have been completed, we can go for a refinement cycle.

Now, we are ready for the refinement of the microstructural parameters. We can proceed in the following fashion: Phases tab → Edit (eye button) → General tab→ Cell parameters →Right-click (on the value 5.4116) → Set to refined. Do not close this window yet. We can also refine the crystallite size and microstrains at this step. So, we can proceed as follows: Switch to Microstructure tab → Size-Strain model → Change from Isotropic to Popa rules→Click on options. The window shown in Fig. S6 will pop up. We should not change any other entity in the microstructure tab. In the Popa model window,

set to refined the _riet_par_anisocryst_size0. Since we already know that the crystallite size is around 125 Å, we can set the default 1000 Å to a lower value. For now, do not refine the _riet_par_anisocryst_size1. However, set to refined the two R.m.s. microstrain terms. Click on "OK". Alternatively, we can perform these steps from the tree structure.

Now we can go for the next refinement cycle. We would notice the changes in the lattice parameter, the crystallite size (size 0), and microstrain values. We should save the file at this point.

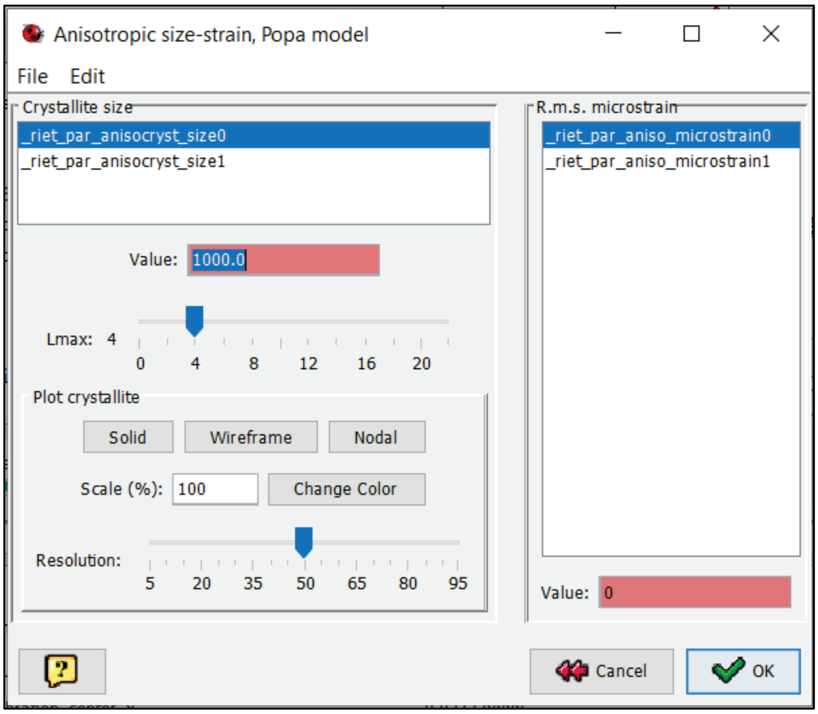


**Figure S6** ImageJ setup for image processing to generate diffraction patterns by azimuthal integration of the 2D image. The red circle is used for aligning the image in the center.

At this stage, if we want to go for Kinematical approximation, we proceed in the following manner: Again, go to the Popa model window as explained before and this time, also set to refined the _riet_par_anisocryst_size1 parameter. However, it is a good idea to start with a non-zero value. So set a value in between 1/2 to 1/3 of the refined _riet_par_anisocryst_size0 value. Click on OK and go for another refinement cycle. We would notice that the intensities fit poorly. To get better results, we can define an overall thermal factor following the steps: Phases → Structure→ right-click on the "Bisofactor" value of Ce1 (see Fig. S7.)→ refined. Since we want the same B factor for both Ce and O, we proceed with refining the B factor of O as follows: Switch to O tab → right-click on the "Bisofactor" value→ select equal to. The window superposed in Fig. S7 pops up. In this window, locate for Ce1 _atom_site_B_iso_or_equiv and select it. Then click on "Set bound" and close this window. Also, close the Cerium Oxide phase window. Go for another refinement cycle. At this step,

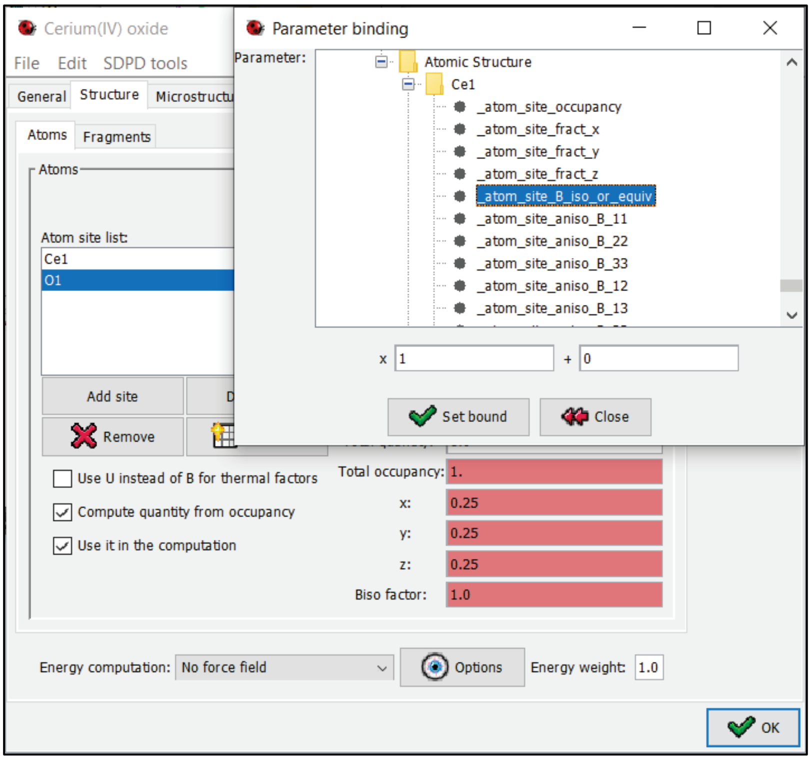


**Figure S7** Setting an equivalent Biso factor for Ce and O.

the analysis under kinematical approximation is completed. We can save this file as CeO2 0012 Ceta_SA200_CL1080_07_two_stage_kin.par

For performing the Le-Bail fitting/Pattern matching, we can proceed as follows: Load the file CeO2 0012 Ceta_SA200_CL1080_07_two_stage_kin.par. We should fix the scale/intensity factors: "_pd_processing_intensity_incident" on the tree structure. We should also fix the overall B-factor refined earlier. So, set to fixed "_atom_site_B_iso_or_equiv" for the Ce1 atom site. Then we should set the texture to arbitrary texture as follows: Cerium oxide→edit (eye button) → Advanced models → Texture → choose arbitrary texture. Click on Ok. Perform another refinement cycle. These would yield the microstructural parameters with Le-Bail fitting.

For obtaining the results with Blackman two-beam correction, we should load the file obtained after refining only _riet_par_anisocryst_size0 crystallite size R.M.S microstrain values. In MAUD, the dynamical correction can be activated in the "Dynamical scattering correction" option under the "Microstructure" tab, with the possibility of using the same anisotropic crystallite size for the line broadening analysis. In the "Grain size (Angstrom)" enter the obtained refined value of _riet_par_anisocryst_size0 and set it to refined. Click on OK. If we now press on "Calculator", we would notice that the experimental and calculated data do not match at all. So, locate the "_pd_processing_intensity_incident" on the tree structure and increase its value till a match is observed. Then, go for a refinement cycle. Finally, refine the _riet_par_anisocryst_size1 and an overall Biso

factor, as done for the Kinematical case. We can save the file as CeO2 0012 Ceta_SA200_CL1080_06_two_stage_dyn.par.

## S8. Procedure to determine amorphous carbon halo contribution

- Load the instrumental setup used as done previously.
- Load the Cu_Grid 0010 Ceta_SA_200um-CL_1080mm.tiff.
- Treat the pattern using the ImageJ plugin as demonstrated previously. Figure S8 shows the electron diffraction image with the shadow of the sample holder removed. After performing the integration, we should save the ASCII files as done for the $CeO_2$ sample. The resulting set of diffractograms is shown in Fig. S9. These are summed up to obtain the 1-D pattern.

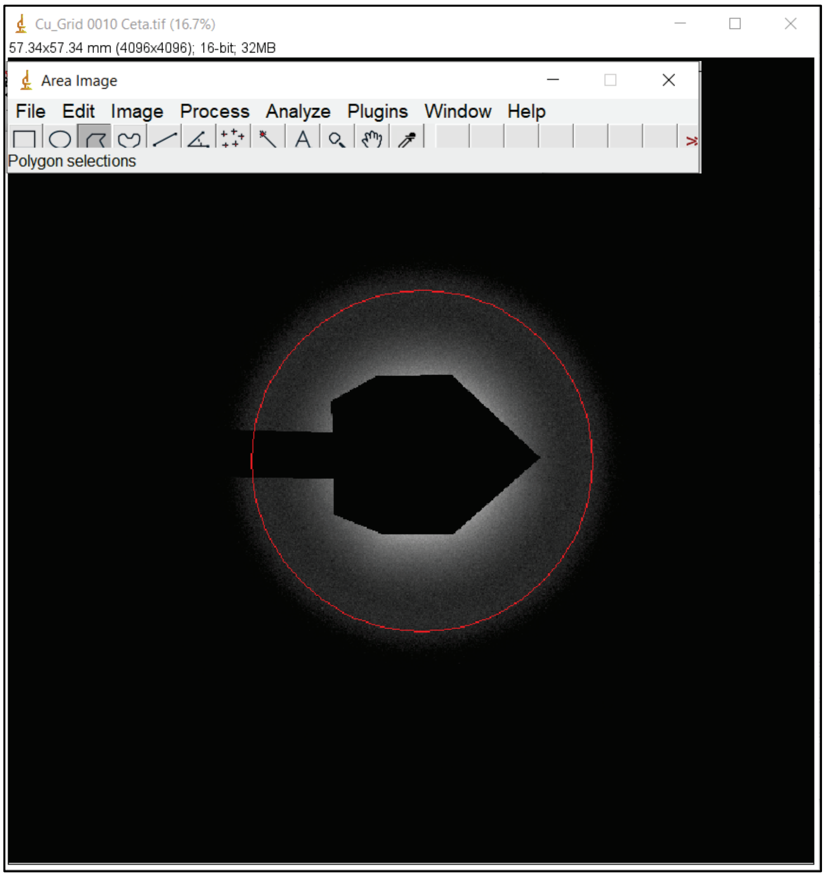


**Figure S8** ImageJ setup for Cu grid diffraction image processing to generate a set of patterns by azimuthal integration of the 2D image. The red circle is used for aligning the image in the center.

- We should add the background peak contribution for the strong signal at 0 Q, as demonstrated in Section S3.
- Open the “Background peaks” section under “Background function”. Click on “add term” and set the initial parameters for the first Halos as follows: Height: 10; position: 0.66; HWHM: 0.07; Eta: 0.26. Set all these values to be refined.

- We repeat the same step for the second Halos and set the Initial values as follows: Height: 10; position: 1.22; HWHM: 0.10; Eta: 0.75. Set all these values to be refined.

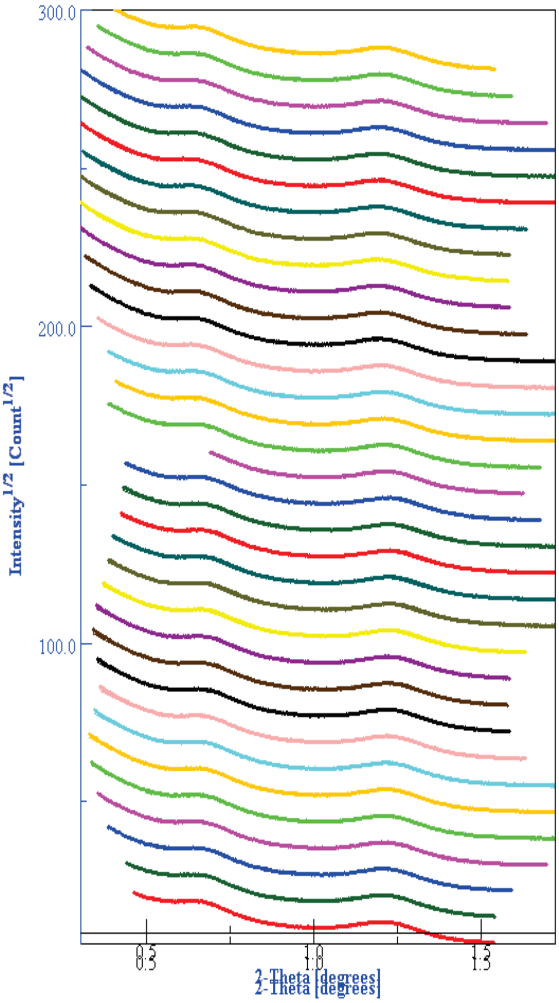


**Figure S9** The resulting set of patterns from the azimuthal integration of the diffraction pattern of the Cu grid shown in Fig. S8

- Then free the scale parameters and background parameters. Run the refinement cycle by pressing on the "Hammer/Machine" icon.

- Collect the refined values of the background contribution from the two Halos. We use these for the background evaluation when we have $CeO_2$ sample on the Cu-grid.
